# Gut Microbiota of Great Spotted Cuckoo Nestlings is a Mixture of Those of Their Foster Magpie Siblings and of Cuckoo Adults

**DOI:** 10.3390/genes9080381

**Published:** 2018-07-27

**Authors:** Magdalena Ruiz-Rodríguez, Manuel Martín-Vivaldi, Manuel Martínez-Bueno, Juan José Soler

**Affiliations:** 1 Biologie Integrative des Organismes Marins, Observatoire Océanologique, Sorbonne Universités, Avenue du Fontaulé, 66650 Banyuls-Sur-Mer, France; magdalenaruizr@gmail.com; 2 Departamento de Zoología, Universidad de Granada, E-18071 Granada, Spain; mmv@ugr.es; 3 Unidad Asociada Coevolución: Cucos, Hospedadores y Bacterias Simbiontes, Universidad de Granada, E-18071 Granada, Spain; mmartine@ugr.es; 4 Departamento de Microbiología, Universidad de Granada, E-18071 Granada, Spain; 5 Departamento de Ecología Funcional y Evolutiva, Estación Experimental de Zonas Áridas (EEZA-CSIC), Ctra. Sacramento s/n, La Cañada de San Urbano, E-04120 Almería, Spain

**Keywords:** brood parasitism, cloaca microbiota, host diet and gut microbiome, host genetic and gut microbiota

## Abstract

Diet and host genetic or evolutionary history are considered the two main factors determining gut microbiota of animals, although studies are scarce in natural populations. The system of great spotted cuckoos (*Clamator glandarius*) parasitizing magpies (*Pica pica*) is ideal to study both effects since magpie adults feed cuckoo and magpie nestlings with the same diet and, consequently, differences in gut microbiota of nestlings of these two species will mainly reflect the importance of genetic components. Moreover, the diet of adults and of nestling cuckoos drastically differ from each other and, thus, differences and similarities in their microbiotas would respectively reflect the effect of environmental and genetic factors. We used next-generation sequencing technologies to analyze the gut microbiota of cuckoo adults and nestlings and of magpie nestlings. The highest α-diversity estimates appeared in nestling cuckoos and the lowest in nestling magpies. Moreover, despite the greatest differences in the microbiome composition of magpies and cuckoos of both ages, cuckoo nestlings harbored a mixture of the Operational Taxonomic Units (OTUs) present in adult cuckoos and nestling magpies. We identified the bacterial taxa responsible for such results. These results suggest important phylogenetic components determining gut microbiome of nestlings, and that diet might be responsible for similarities between gut microbiome of cuckoo and magpie nestlings that allow cuckoos to digest food provided by magpie adults.

## 1. Introduction

The study of symbiotic associations between macro- and microorganisms has become one of the most fruitful areas of biological research during the last decade [1]. This is mainly because symbiotic relationships between animals and microorganisms can have a profound impact on animal evolution, among other reasons, because microbes that are integrated within the hosts can perform essential functions for them, while hosts provide particular environments that are essential for microbes’ reproduction and dispersal [2,3]. It has even been suggested that considering host and microbiome as a single ecological unit (the holobiont) would facilitate the understanding of evolutionary processes [4]. However, microbes do not colonize animals for the benefit of the hosts but rather to take advantage of available food or habitat resources [5,6]. Whatever the underlying cause and its implication for evolutionary theory, characterizing the microbial diversity associated with different hosts, as well as possible functionalities for them, is essential for understanding animal adaptation to environmental conditions.

The majority of bacteria associated with animals are found within the gut, which influences the host physiology, immunity, and development [3,7]. Thus, the gut microbiota of animals is greatly beneficial for hosts’ life, and exploring factors affecting interspecific variation, as well as microbiota acquisition, stability, and homeostasis, would help to understand the role of microorganisms in animals’ functioning and evolution [8]. Host-related environmental factors are considered to play a major role in explaining intra- and interspecific variation in gut microbiota [9,10]. Host diet determines the nutritive environment for bacterial growth within the gut of hosts and is considered the most important environmental factor explaining gut microbiota [3,11,12,13], while the immune system of hosts is also important [14,15]. This is because the immune system constitutes the main host defense against microbial infections [16], but also because it includes components (i.e., IgA) that enhance mucosa colonization by particular beneficial microorganisms [14,17]. Host genetic or evolutionary history are also important factors determining host gut microbiota [3,18], which has recently been demonstrated in laboratory tests. In short, by using animals of different sister taxa that were bred in captivity for more than 10 generations, and by maintaining them on the same diet, Kohl et al. [19] found that gut communities were highly distinguishable by host species.

The vast majority of research on intra- and interspecific variation in gut microbiotas, and on their underlying factors, has been performed in mammals, particularly in humans and captive animals from farms, laboratories or zoos [3]. Inferences drawn from these studies performed in controlled settings might, however, be biased because microbiomes are strongly influenced by different environmental factors, which might interact with each other [20]. A better understanding of factors determining gut microbiotas of animals will, therefore, require conducting research on wild animals in natural conditions [3,20]. Colston and Jackson [3] recently reviewed studies performed with different non-mammalian vertebrate taxa in the wild and highlighted the need for a wider taxonomic sampling of natural rather than captive hosts.

In an attempt to contribute to filling this gap, we describe here gut microbiota of adults and nestlings of the brood parasitic great spotted cuckoo (*Clamator glandarius*) and of nestlings of their main host species in Europe, the magpie (*Pica pica*), in a natural setting. Avian brood parasite–host systems offer important advantages to exploring factors affecting the microbiome of animals [21]. Interspecific brood parasites lay their eggs in nests of other species, the hosts, which incubate the eggs and feed the offspring during the nestling and fledging stage [22]. On the one hand, exploring differences between brood parasite nestlings reared in nests of different host species allows exploring the effect of diet on the gut microbiota in natural environmental conditions. On the other hand, in brood parasite–host systems where both host and parasite nestlings are reared by the host with similar diets (such as magpie–great spotted cuckoo), detecting differences in gut-microbiota characteristics of brood-parasite and host nestlings should be interpreted as evidence of genetic components (i.e., interspecific difference in evolutionary trajectories) determining gut microbiota of birds. Furthermore, it is also common that diets of brood parasitic nestlings greatly differ from diets during adulthood, which should influence gut microbiota of these two stages. Gut microbiota that should fit juvenile and/or adult requirements is acquired during growth [23,24]. In the case that diet of brood-parasites drastically change from nestling to adult, microbiome of brood parasitic nestlings might adapt the requirements of either nestlings or adults, but in the second case, that of nestlings would be suboptimal [25]. Comparison between gut microbiota of brood parasitic adults and nestlings is, therefore, important for exploring the possibility that gut microbiota adapts to different stages.

Particularities and previous knowledge on the great spotted cuckoo–magpie system makes it ideal to study microbiome of hosts and brood parasites in a comparative framework. We know for instance that the diet of cuckoo and magpie nestlings do not differ significantly [26] and, thus, possible differences between gut-microbiota of nestlings of these species would hardly be interpreted as a consequence of diets. Moreover, in comparison with magpie nestlings, great spotted cuckoo nestlings suffer less from parasitism and demonstrate stronger immune response [27]. Thus, since immunity and parasitism are believed to affect the gut-microbiota of animal hosts [14,16,17,28], interspecific differences in the gut microbiota of great spotted cuckoo and magpie nestlings can be predicted. Partially in accordance with that prediction, Ruiz-Rodríguez et al. [25] detected significant differences in bacterial assemblages of the gut microbiota of great spotted cuckoo and magpie nestlings, but they did not detect specific phylotypes in parasitic and host nestlings. Moreover, we also know that magpie and cuckoo nestlings sharing a similar microbiome community at the cloaca, also present the same level of immune response (in magpies) and of body condition (in cuckoos) [29]. Gut microbiota of great spotted cuckoos and magpie nestlings in these previously published works were characterized by means or ribosomal intergenetic space analyses (RISA), with no information on the identity of microbial taxa. Here, we used next-generation sequencing technologies (NGS), which allow affordable sequencing at the deepness needed to sufficiently characterize the diverse bacterial communities [30,31]. We also know that the diet of great spotted cuckoos during the nestling and adult stage differs. Adult great spotted cuckoos almost exclusively feed on caterpillars and are specialist consumers of noxious insects that most birds avoid [32]. Instead, during the nestling phase, they are fed mainly with a variety of insects (larvae and adults), but their diets include fruits and even cereal seeds [33]. Thus, comparing gut microbiota between adults and nestlings is particularly interesting for great spotted cuckoos because it might shed light on particular bacterial groups associated with these changes in cuckoo diets. We did so in this paper and analyzed gut microbiota of magpie and great spotted cuckoo nestlings and of adult cuckoos using NGS (Illumina MiSeq). The above theoretical scenario would allow interpreting expected differences as the result of evolutionary history (i.e. phylogeny). It is assumed that birds acquire gut microbiota during the nesting stage from nest environment and from food consumed [34]. We also know that lifestyle and ontogeny influence gut-microbiota composition throughout associated changes in the environmental conditions (e.g., diet) and physiology (e.g., immunity) [3,11,35,36]. Thus, differences between gut-microbiota of nestling and adult cuckoos could be interpreted as mainly due to change in lifestyle (i.e., diet).

## 2. Materials and Methods

### 2.1. Study Area and Field Work

The study was performed in the Hoya de Guadix (37°18′ N, 3°11′ W), southern Spain; a high-altitude plateau where magpie nests parasitized by great spotted cuckoo are common [37]. The vegetation is scarce with oak (*Quercus rotundifolia*), almond trees (*Prunus dulcis*), and pines (*Pinus halepensis*) in which magpies nest at a relatively high density. Subpopulations are separated by large extensions of arable lands with few or no potential nest sites for the magpies [38].

At the beginning of the breeding season of 2011, we searched for magpie nests and determined the laying date (by assuming a single magpie egg was laid daily), the start of incubation (usually with the fourth egg), and parasitism by the great spotted cuckoo. This information allowed the estimation of the expected date of hatching (21 days and 15 days after the fourth egg was laid for magpie and great spotted cuckoo eggs, respectively). Great spotted cuckoos typically abandon the nest 18–19 days after hatching, while nestling period of magpies extends 4–5 days more. Thus, we sampled great spotted cuckoo and magpie nestlings at the age of 16 and 18 days respectively, which corresponds to similar developmental stage in the two species [39].

Adult cuckoos were caught during the egg-laying stage by mean of mist-nets that we assembled in feeding areas (pine plantations). We used recorded great spotted cuckoo voices played close to the mist nets to attract cuckoo adults.

Samples of gut microbiota were collected in the field by injecting and repipetting 500 µL of sterile phosphate buffer (Na_2_HPO_4_ 0.1 M and NaH_2_PO_4_ 0.1 M, pH 7.4) in the cloaca using sterile tips and an automatic pipette. After collection, we immediately lysed the bacterial cells by adding 500 µL of lysis buffer (5% Tris/HCl 50 mM, 5% SDS, EDTA 2 mM, NaCl 100 mM) and samples were kept in ice [25,29,40]. Later in the lab, samples were stored at −20 °C until molecular analyses. We sampled 6 adult great spotted cuckoos, 7 magpie nestlings from non-parasitized nests, and 12 cuckoo nestlings from parasitized nests. Only one nestling per nest was sampled.

### 2.2. Ethics Statement

The study was conducted according to relevant Spanish national (Decreto 105/2011, 19 Abril) and regional guidelines. All necessary permits for bird manipulations were provided by Consejería de Medio Ambiente de la Junta de Andalucía, Spain (Ref: SGYB/FOA/AFR). Our study area is not protected but privately owned, and the owners allowed us to work on their properties. The time spent in each magpie nest was the minimum necessary for sampling one of the nestlings. This manipulation did not show detectable effects in nestlings.

### 2.3. Laboratory Work

DNA was extracted from 200 µL of each sample containing lysis buffer. Samples were thermically shocked to further lyse the cells, and then DNA was extracted by using the PowerSoil DNA Isolation Kit (Mo BIO, Carlsbad, CA, USA) following the manufacturer’s instructions. We did, however, change the last step. Instead of adding 100 µL of elution buffer and centrifuge immediately, we added 50 µL and incubated for 1 min at room temperature before centrifuge. Then, we repeated with other 50 µL of the elution buffer. It was done to maximize the quantity of DNA that we could recover from the filter.

Samples were amplified according to the Earth Microbiome Project (EMP) protocol [41], producing approximately 300 bp of the 16S ribosomal DNA (rDNA) V4 hypervariable region. Two negative controls of the extraction procedure and one blank control for PCR amplification was included in the sequencing. The libraries were then sequenced in a single run of IlluminaMiSeq (2 × 300 bp output mode) sequencer at Integrated Microbiome Resource, Centre for Comparative Genomics and Evolutionary Bioinformatics (CGEB), University of Dalhousie (Canada). The universal primers 515F and 806R [42,43] were modified to include part of the Illumina adapters (and a 5 bp barcode in the forward primer for multiplexing) as follows: U515F (5′-ACACGACGCTCTTCCGATCT-NNNNN-GTGCCAGCMGCCGCGGTAA-3′) and E806R (5′-CGGCATTCCTGCTGAACCGCTCTTCCGATCT-GGACTACHVGGGTWTCTAAT-3′).

### 2.4. Sequence Data Analysis and Taxonomic Identification

The 16S rDNA variable region 4 (V4) was sequenced in Illumina MiSeq 2000 Platform. Sequences are available in NCBI Repository (BioProject PRJNA482612, accession: http://www.ncbi.nlm.nih.gov/bioproject/482612). Sequence processing to get an Operational Taxonomic Unit (OTU) table was performed following QIIME software v1.9.1 (Quantitative Insights In Microbial Ecology; [44]) and recommendations on genomic data processing [45,46,47]. Briefly, sequences of paired-read amplicon libraries were paired-end aligned using fastq-join method [48], with a minimum overlap of 100 base-pairs and maximum 10% difference in the overlapping region. Then demultiplexion and quality filtering (at Phred ≥ Q20) was performed, and sequences trimmed to 400 base-pairs with Usearch [49]. The subsampled open-reference OTU picking procedure [50] was applied to generate an OTU table, clustering sequences against Greengenes database v 13.8 at 97% of similarity [51,52], with a minimum OTU size of 10 sequences, the reverse-strand-match option enabled and suppressing step 4 (a second round of de-novo picking). Results did not change when using OTU size of 2 sequences instead. Subsequently, the OTU table was filtered to remove Archaea, chloroplasts, mitochondria, non-phylum assigned OTUs, singletons, and OTUs with a frequency lower than 0.005% of the total sequence account [53]. To control for the sequencing effort, we performed a multiple rarefaction (10 random repetitions) at 3300 sequences and performed analyses with each of the rarefied files to obtain mean (SE) estimates of the parameters of the statistical models applied.

### 2.5. Statistics

QIIME, the bioinformatics pipeline for performing microbiome analysis from raw DNA sequencing data, was used to generate α-diversity estimates for samples (richness (number of OTUs) and Shannon index), and β-diversity matrices of distances among samples (unweighed and weighed UniFrac distances [54]). Comparisons among types of samples were performed with Primer7 and Statistica 13.0 [55]. Comparisons of α diversity estimates were performed using ANOVAs and post-hoc Tukey tests. These variables did not differ from Gaussian distributions (Kolmogorov Smirnov tests: all *p* > 0.2) and there was homogeneity of variances among groups (Levene tests, number of OTUs: F(2,22) = 1.03, *p* = 0.372; Shannon index F(2,22) = 1.99, *p* = 0.161). The composition of the microbiota was compared among types of samples using PERMANOVAs and post-hoc pair-wise *t*-tests in Primer7 [56]. Briefly, we used (a) weighed and unweighed UniFrac distance matrices generated with QIIME for the OTU level, or (b) Bray Curtis (abundance) and Jaccard (presence-absence) resemblance matrices calculated in Primer7 for the summary table of sequences at the family level generated in QIIME. Principal Coordinates analyses (PCo) were used to visualize the relative position of the three types of samples in the multidimensional space of microbiome composition. Stepwise BEST analyses (1000 restarts) run in Primer7 were used to select the best combination of families explaining differences in microbiome composition among types of samples, and the families included in the best four models were tested for differences in abundance (with ANOVAs) and prevalence (with Pearson Χ^2^ tests) among sample types. The main analyses were conducted independently with the 10 rarefied OTU tables and mean (SE) values of parameters are presented. The post-hoc analyses were performed only with the first rarefaction.

## 3. Results

**Figure 1:**
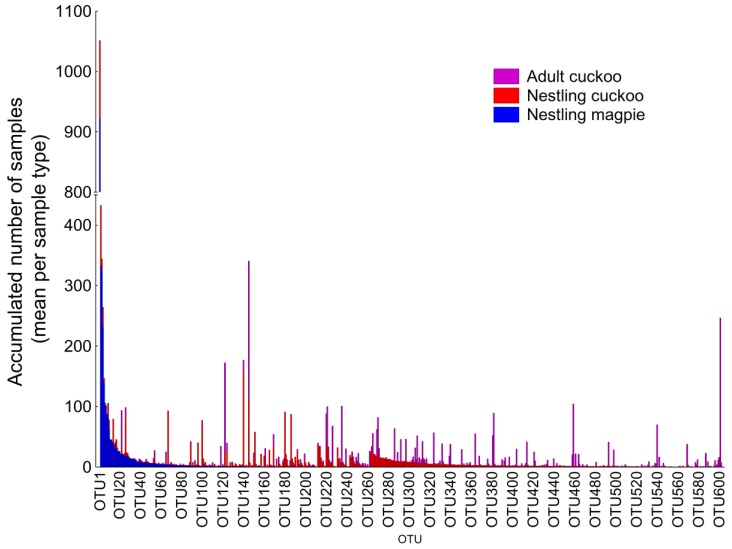
Differences in the abundance of the different Operational taxonomic Units (OTUs) (mean number of sequences per sample) in the cloaca of magpie nestlings, cuckoo nestlings, and cuckoo adults. Bars represent the accumulated number of sequences and OTUs are ordered by their abundance in magpies, and afterward in nestling cuckoos. Only OTUs with at least 25% of prevalence in any sample type are considered.

The Illumina analysis detected 741 different OTUs considering all the three types of samples obtained from magpie and great spotted cuckoos (hereafter cuckoos) (see Supplementary Materials). Most of those OTUs (81.2%) were present in at least 25% of samples of one or more of the sample types (Supplementary Materials, Figure S1). Significantly, the cloacae of nestling cuckoos presented the highest bacterial richness compared with those of nestling magpies or adult cuckoos while the bacterial richness of magpie nestlings and of adult cuckoos did not differ significantly (Table 1). There were, however, clear differences in the particular OTUs prevailing in magpie samples and those of cuckoos, with several OTUs that are highly abundant in magpie samples but scarce or absent in cuckoo samples, and a majority of OTUs that were detected in cuckoo samples that were not detected in those of magpies (Figure 1). Thus, the α-diversity estimates for the three groups of samples differed significantly (Table 1). The highest α-diversity estimates appeared in nestling cuckoos and the lowest in nestling magpies, while those of adult cuckoos were intermediate (Table 1).

**Figure 2:**
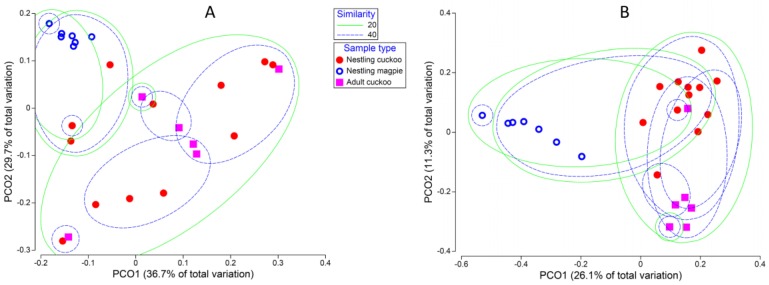
Differences in whole composition of the cloaca microbiota among cuckoo nestlings, magpie nestlings, and adult cuckoos. (**A**) Distances based on weighed unifrac β-diversity; (**B**) Distances based on unweighed unifrac β-diversity. Lines surround samples clustered by percentage of similarity (20% or 40%) among their whole microbial communities. PCO2: second principal coordinate axis; PCO1: first principal coordinate axis.

The microbiota composition of the three types of samples differed significantly (Table 1). Magpie samples differed from those of cuckoos (either, nestlings and adults) with respect to all β-diversity estimates. Differences between adult and nestling cuckoos were more patent when considering unweighed unifrac β-diversity (Table 1, Figure 2), which suggests that the less abundant OTUs are relatively more important in explaining such detected differences. When considering community similarities, adult cuckoos and nestling magpies almost never clustered together. However, some (weighed unifrac) or most nestling cuckoos (unweighed unifrac) clustered with both magpies and adult cuckoos, showing that their microbiomes are a combination of OTUs of the other two sample types (Figure 2). The results were very similar when considering only those OTUs present in at least 25% of any sample type (results not shown).

**Figure 3:**
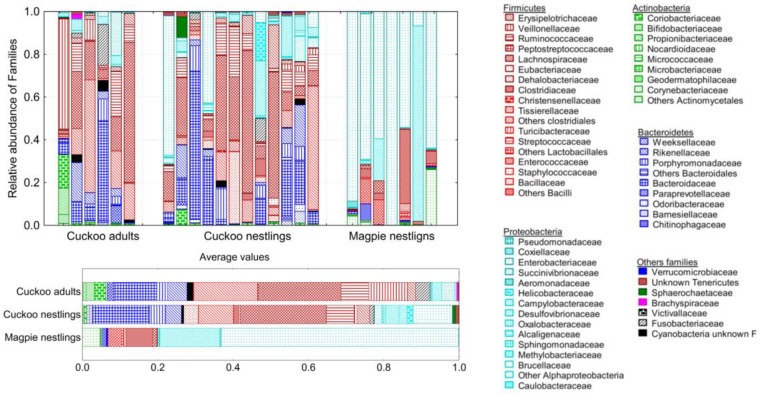
Relative abundance of bacterial families belonging to different phyla detected in the gut microbiome of cuckoo adults, cuckoo nestlings, and magpie nestlings. Individual and average information are shown.

Differences in bacterial composition of different types of samples were quite evident even at the highest taxonomic levels, with a clear dominance of phylum Proteobacteria in magpies and of Firmicutes and Bacteroidetes phyla in cuckoos (Figure 3). At the taxonomic level of Family, the best subset of models explaining differences in relative abundance of bacteria (Bray-Curtis models) among the three sample types included six families (Table 2, Figure 3). Comparisons of the abundance of these six families among cuckoo (nestlings and adults) and magpie samples only reached statistical significance for Ruminococcaceae (more abundant in cuckoos) and Enterobacteriaceae (more abundant in magpies) (Table 2). When considering information of bacterial presence (Jaccard models), 16 families entered in the best subsets, many of them significantly prevailing in either cuckoos or magpies, and some of them differing between adult and nestling cuckoos (Figure 3, Table 2). Four families of the phylum Bacteroidetes (Rikenellaceae, Odoribacteriaceae, Barnesiellaceae, and Paraprevotellaceae) were significantly more prevalent in cuckoos, three of them being completely absent from magpies. The same was true for two families of phylum Proteobacteria (Alphaproteobacteria and Alcanigenaceae) and the phylum Cyanobacteria, which were not detected in magpie samples (Table 2). One additional family of Proteobacteria (Succinivibrionaceae) was only detected in adult cuckoos (Table 2). Three families of the phylum Firmicutes (“other” clostridiales, Veillonellaceae and Erypsipelotrichaceae) were more frequent in cuckoos than in magpies, two families (“other” lactobacillales and Enterococcaceae) were more frequent in magpies than in cuckoos, and the family Clostridaceae was more frequent in nestlings (magpies or cuckoos) than in adult cuckoos (Table 2). Interestingly, the prevalence of these three last families in nestling cuckoos were intermediate between magpies and adult cuckoos (Table 2). Finally, two families in the phylum Actinobacteriaceae (Nocardioidaceae and Micrococcaceae) were more common in magpies than in cuckoos (Table 2, Figure 3).

## 4. Discussion

Our main findings are twofold: One refers to detected differences between gut microbiota of magpies and cuckoo nestlings; the other states that gut microbiota of nestling cuckoos is a mixture of those of adult cuckoos and magpie nestlings, two phylogenetically distant species. The first group of results suggests an important phylogenetic component determining gut microbiome of nestlings, while the second would be consistent with an interpretation of functional differences. Cuckoo nestlings would acquire bacteria while growing in nests of magpies that are useful to digest food provided by adult hosts, but also others that will be useful for cuckoos during the adult stage. Below we discuss the importance of these results in the understanding of the role of genetic, environmental, and ontogenetic factors determining gut microbiota of animals, as well as possible bacterial sources for nestling cuckoos explaining the detected differences.

Differences in the microbiome of magpie and great spotted cuckoo nestlings were detected in terms of bacterial richness, α-diversity, community composition, and abundance and prevalence of bacteria at different taxonomic levels. Consistent with previous papers characterizing gut microbiota of birds [3,35], those of cuckoos and magpies were dominated by members of the phyla Firmicutes, Proteobacteria, Bacteriodetes, and Actinobacteria. However, Firmicutes and Bacteriodetes were more abundant in gut microbiotas of cuckoos, and Proteobacteria were more abundant in those of magpies. Most of the detected Bacteriodetes families were absent in magpies’ microbiota. Ruminococcaceae (Phylum Firmicutes) were more abundant in the gut microbiota of cuckoos, while Enterobacteriacea (Phylum Proteobacteria) was more abundant in that of magpies. We also detected interspecific differences in the prevalence of many of the detected bacterial families, or even Operational Taxonomic Units (Supplementary Materials). Our knowledge on the functionality of different bacterial groups on the digestion process of birds is, however, quite limited, and we can only speculate that differences between adult and nestling cuckoos, as well as similarities between cuckoo and magpie nestlings, allow developing cuckoos to process the food provided by magpie foster parents. Further research, including experimental manipulation of diets, is necessary for exploring possible functionalities of cuckoo and magpie gut microbiotas.

Gut microbiota of birds may be acquired from parents or from the environments; host diets determine the nutritive environment for bacterial growth and, thus, is considered the most important environmental factor determining gut microbiota [3]. Interspecific brood parasites develop in nests of other species and have no contact with brood parasitic adults during the nestling phase of development. Thus, possibilities of vertical transmission of microorganisms from adult to nestling cuckoos are very limited, which allows the exploration of the effect of diet in organisms of different evolutionary history in natural conditions (i.e., taxonomy) [21,25]. Hird et al. [21] did not detect interspecific differences between the gut microbiota of the brood parasitic brown-headed cowbird (*Molothrus ater*) and that of their main hosts. They, therefore, concluded that the detected interspecific variation was mainly environmentally determined. Brown headed cowbirds are phylogenetically closely related to their hosts (passerines), and both have similar diets [57]. Therefore, it is possible that optimal gut microbiota of cowbird and their hosts was also similar and dependent on environmental conditions [21]. Differing from cowbirds, great spotted cuckoos are within the subfamily Cuculidae, and their diets greatly contrast with that of their hosts, which are also passerines [57]. In accordance with differences in phylogenetic position and diet between magpie and great spotted cuckoo, Ruiz-Rodríguez et al. [25] detected interspecific differences in the gut-microbiota of great spotted cuckoo and of magpie nestlings, and we here labeled bacterial taxa responsible for such differences. These results strongly suggest a phylogenetic component of gut microbiota of magpies and great spotted cuckoos that are independent of the diet received during the nesting phase.

Several mechanisms may explain the detected differences between magpies and great spotted cuckoo nestlings. Nestlings of the two species are fed with similar food [26,33] and, thus, diet cannot be responsible for detected differences. Interspecific differences in physiological, anatomical, or life history characteristics of animals are linked to their gut microbiota [58] and, thus, these characteristics might be responsible for the detected differences. Great spotted cuckoo and magpie nestlings differ, for instance, in the morphology of their intestine, with only that of cuckoos presenting a relatively large caeca, a pair of appendages protruding from the junction of the small and the large intestine [25]. Caeca harbor special microbiota that facilitate nitrogen cycling, carbohydrate fermentation, and aid water retention [59,60,61] and, thus, may be responsible for detected interspecific differences. Great spotted cuckoo nestlings also have stronger immune responses, suffer less from ecto- and blood-parasites and from costs related to sibling competition than magpie nestlings (see Introduction). All these factors are usually related to gut-microbiome characteristics. The study of intraspecific covariation between gut-microbiota characteristics and such factors would help to figure out the relative importance of each of them.

Gut-microbiota of cuckoo nestlings was more similar to that of cuckoo adults than to the gut microbiome of magpie nestlings. This result suggests that the gut microbiome of cuckoo nestlings is prepared for processing typical prey that they will consume during the adult phase. How do cuckoo nestlings manage to incorporate in their gut-microbiome components that do not appear in the gut of their foster siblings but that will be useful during the adult stage? Two mechanisms may allow nestling cuckoos to recruit such bacteria. The first one is that particularities of the intestine of cuckoos favor colonization and growth of particular bacteria that may not be the optimal for digesting food received during the nestling phase but for digesting prey consumed during the adult phase (see, [25]). If we assume that most of the microorganisms in the gut of their avian host come from the environment [3] (i.e., the gut of their (foster) parents and the microbiome of the consumed prey items), particular morphology and biochemical characteristics of great spotted cuckoo intestine might be adapted to favor those that maximize fitness components, not only during the nestling phase, but also during the adulthood.

Vertical transmission of microbial symbionts from female cuckoos during egg formation and/or laying might also explain the detected similarities in the gut microbiome of nestling and adult cuckoos. Although physical contacts between nestling and adult cuckoo are possible, it mainly takes place during the fledgling stage and, to a lesser extent, at the end of the nestling period [62]. We sampled nestling cuckoos at the end of the nestling period and, thus, it is unlikely that the occasional contacts during this stage explained the detected similarities in gut microbiotas. Another possibility is that female cuckoos transfer symbiotic microorganisms to the eggs during egg formation or during laying when crossing the cloaca. It has been suggested recently that eggs may function as transgenerational carriers of the maternal microbiota [63]. Previous results exploring bacterial communities of the eggshells of great spotted cuckoos and of magpies within the same magpie nests revealed statistically significant differences for some bacterial groups [64], which may suggest that these differences might be mediated by characteristics of the gut microbiota of female cuckoos. As far as we know, the association between the microbiota of the eggshells and of the cloaca of laying females has been explored in European hoopoes (*Upupa epops*) [40] and in two species of larks [63]. In no case did bacterial communities of the eggshells reflect the gut microbiome of females, which provides little support for the hypothetical role of eggshell microbiota. In any case, further work exploring the association between the microbiota of the cuckoo eggshells, and that of the cloaca of cuckoo females, is necessary for further rejecting this possibility.

## 5. Conclusions

Whatever the mechanisms, our findings indicating that characteristics of the microbiome of nestling cuckoos are intermediate between those of magpies and of adult cuckoos, strongly suggests a role for phylogeny determining gut-microbiome of birds that are independent of the diet received during development. Diet might be responsible for similarities in the gut microbiome of cuckoo and magpie nestlings that allow cuckoos a proper digestive gut-microbiome.

## Figures and Tables

**Table 1:** Comparisons of gut microbiome of magpie and great spotted cuckoo nestlings and of great spotted cuckoo adults in terms of bacterial richness, α-diversity, composition (weighed and unweighed unifrac β-diversity) and abundance and prevalence of bacterial families.

VARIABLE CONSIDERED	TYPE OF SAMPLE			COMPARISONS				
	(A)	(B)	(C)	ANOVA		POST-HOC (Tukey tests)		
	Adult cuckoos	Nestling cuckoos	Nestling magpies					
	(*N* = 6)	(*N* = 12)	(*N* = 7)			A vs. B	A vs. C	B vs. C
	Mean	Mean	Mean	F_2,22_	*p*	*p*	*p*	*p*
	(SE)	(SE)	(SE)	(SE)	(SE)			
Bacterial Richness	73.8	123	52.29	14.42	**0.0001**	**0.008**	0.405	**0.0003**
	−12.1	−8.5	−11.2	−0.19	**−0.000008**			
α-diversity (Shannon index)	3.92	4.45	2.66	6.5	**0.006**	0.585	0.106	**0.005**
	−0.43	−0.31	−0.4	−0.03	**−0.00001**			
				PERMANOVA		POST-HOC (Pair-wise *t*-tests)		
Microbiome composition				Pseudo-F (SE)	*p* (SE)	*p* (*t*-value)	*p* (*t*-value)	*p* (*t*-value)
Weighed unifrac β-diversity				4.35	**0.00022**	0.559	**0.0002**	**0.0001 (2.63)**
				−0.01	**−0.00004**	−0.85	**−2.87**	
Unweighed unifrac β-diversity				5.37	**0.0001**	**0.0002**	**0.0007**	**0.0001**
				−0.07	**−0.0001**	**−1.72**	**−2.47**	**−2.61**
Abundance of Families (Bray−Curtis distance matrices)				5.33	**0.00013**	0.58	**0.0008**	**0.002 (2.94)**
				−0.03	**−0.00002**	−0.9	**−3**	
Prevalence of Families (Jaccard distance matrices)				6.43	**0.0001**	**0.0034**	**0.0006**	**0.0001 (2.86)**
				−0.12	**−0.00001**	**−1.62**	**−2.55**	

Bold fonts highlight statistically significant results (*p* < 0.05).

**Table 2:** Bacterial families that entered in the four Bray-Curtis best models (Model 1: r = 0.954, Model 2: r = 0.959, Model 3: r = 0.959, Model 4: r = 0.955) or in the four Jaccard best subject models (Model 1: r = 0.955, Model 2: r = 0.952, Model 3: r = 0.952, Model 4: r = 0.952). Mean values of abundance ± standard error (SE) of families that entered in Bray-Curtis best subset models of samples from adult cuckoos and from cuckoo and magpie nestlings are provided, as well as the results from comparisons (ANOVAs) between the three types of samples. Similarly, prevalence (%) of families that entered in the Jaccard best subset models are shown for samples from adult cuckoos and from cuckoo and magpie nestlings, as well as the results from comparisons (Pearson χ^2^) between the three types of samples.

		Using Information of Microbial Abundance						Using Information of Microbial Prevalence					
		Bray-Curtis	Mean(SE)			ANOVAs		Jaccard	Prevalence (%)			Pearson χ^2^	
Phylum	Family	Entered Best Models	Adult Cuckoos (*N* = 6)	Nestling Cuckoos (*N* = 12)	Nestling Magpies (*N* = 7)	F(_2,22_)	*p*	Entered Best Models	Adult Cuckoos (*N* = 6)	Nestling Cuckoos (*N* = 12)	Nestling Magpies (*N* = 7)	χ^2^	*p*
Actinobacteria	Bifidobacteriaceae	1	0.022 (0.020)	-	-	1.931	0.169						
	Corynebacteriaceae							1–4	50.00	75.00	71.43	1.201	0.548
	Propionibacteriaceae							1–3	**100.00**	**66.67**	**100.00**	**5.159**	**0.076**
	Nocardioidaceae							1, 4	**16.67**	**0.00**	**42.86**	**6.045**	**0.049**
	Micrococcaceae							4	**0.00**	**0.00**	**42.86**	**8.767**	**0.012**
Bacteroidetes	Bacteroidaceae	1–4	0.113 (0.072)	0.148 (0.058)	0.003 (0.002)	1.732	0.2						
	Porphyromonadaceae							1–4	83.33	83.33	42.86	4.096	0.129
	Rikenellaceae							1–4	**83.33**	**83.33**	**0.00**	**14.583**	**<0.001**
	Odoribacteriaceae							2, 3	**33.33**	**66.67**	**0.00**	**8.333**	**0.016**
	Barnesiellaceae							1–4	**83.33**	**58.33**	**14.29**	**6.542**	**0.038**
	Paraprevotellaceae							4	**66.67**	**16.67**	**0.00**	**8.553**	**0.014**
Cyanobacteria	Cyanobacteria							1–4	**66.67**	**75.00**	**0.00**	**10.644**	**0.005**
Firmicutes	Lachnospiraceae	1–4	**0.220** **(0.095)**	**0.228** **(0.065)**	**0.012** **(0.008)**	**3.036**	**0.069**						
	Other clostridiales	2, 3	0.169 (0.103)	0.093 (0.046)	0.006 (0.005)	2.083	0.148	1, 3	**100.00**	**100.00**	**42.86**	**12.245**	**0.002**
	Ruminococcaceae	1, 3, 4	**0.074** **(0.034)**	**0.074** **(0.019)**	**0.00004** **(0.00004)**	**3.547**	**0.046**						
	Clostridiaceae	1–4	0.002 (0.001)	0.014 (0.007)	0.070 (0.047)	2.146	0.141	1–4	**33.33**	**91.67**	**85.71**	**7.965**	**0.019**
	Veillonellaceae	2, 3	0.103 (0.084)	0.008 (0.004)	0	2.251	0.129	1–4	**66.67**	**66.67**	**0.00**	**8.974**	**0.011**
	Erypsipelotrichaceae							2	**100.00**	**100.00**	**28.57**	**16.071**	**<0.001**
	Staphyllococcaceae							4	50.00	50.00	42.86	0.103	0.95
	Other Lactobacillales							2, 3	**16.67**	**33.33**	**85.71**	**7.317**	**0.026**
	Eubacteriaceae							1, 2	16.67	25.00	0.00	2.059	0.357
	Enterococcaceae							1–4	**0.00**	**16.67**	**71.43**	**9.647**	**0.008**
Proteobacteria	Enterobacteriaceae	1–4	**0.005** **(0.003)**	**0.104** **(0.061)**	**0.630** **(0.112)**	**17.447**	**<0.001**						
	Campylobacteraceae	1, 2	0	0.021 (0.021)	0.159 (0.129)	1.555	0.233						
	Helicobacteriaceae	4	0	0.015 (0.015)	0	0.526	0.598						
	Other Alphaproteobacteria							1	**50.00**	**91.67**	**0.00**	**15.192**	**<0.001**
	Pseudomonadaceae							2	**0.00**	**25.00**	**57.14**	**5.336**	**0.069**
	Brucellaceae							4	16.67	16.67	0.00	1.326	0.515
	Succinivibrionaceae							1	**50.00**	**0.00**	**0.00**	**10.795**	**0.0045**
	Alcanigenaceae							3, 4	**33.33**	**75.00**	**0.00**	**10.457**	**0.0054**
Verrucomicrobia	Verrucomicrobiaceae							3	16.67	41.67	0.00	4.441	0.109

Comparisons with associated *p*-values lower than 0.1 are highlighted in bold font.

## Supplementary Materials

The following are available online at http://www.mdpi.com/2073-4425/9/8/381/s1, Table S1: Comparison of the abundances of each particular OTU among the cloacae samples obtained from adult and nestling great-spotted cuckoos as good as nestling magpies, Table S2: List of OTUs with a prevalence higher than 25% in any of the three types of samples, Figure S1: Tree reflecting the among-otus phylogenetic relationships for OTUs found in cloacae of great-spotted cuckoos and magpies.
