# Correction: Ruiz-Rodríguez et al. Gut Microbiota of Great Spotted Cuckoo Nestlings Is a Mixture of Those of Their Foster Magpie Siblings and of Cuckoo Adults. *Genes* 2018, *9*, 381

**DOI:** 10.3390/genes9110530

**Published:** 2018-10-31

**Authors:** Magdalena Ruiz-Rodríguez, Manuel Martín-Vivaldi, Manuel Martínez-Bueno, Juan José Soler

**Affiliations:** 1 Biologie Integrative des Organismes Marins, Observatoire Océanologique, Sorbonne Universités, Avenue du Fontaulé, 66650 Banyuls-Sur-Mer, France; magdalenaruizr@gmail.com; 2 Departamento de Zoología, Universidad de Granada, E-18071 Granada, Spain; mmv@ugr.es; 3 Unidad Asociada Coevolución: Cucos, Hospedadores y Bacterias Simbiontes, Universidad de Granada, E-18071 Granada, Spain; mmartine@ugr.es; 4 Departamento de Microbiología, Universidad de Granada, E-18071 Granada, Spain; 5 Departamento de Ecología Funcional y Evolutiva, Estación Experimental de Zonas Áridas (EEZA-CSIC), Ctra. Sacramento s/n, La Cañada de San Urbano, E-04120 Almería, Spain

The authors wish to make the following changes in their paper [1]. The hypervariable DNA region we sequenced was the V6-V8 rather than the V4 as we erroneously wrote in the printed version. For the corrected version, we have added information on the universal primer used, as well as the performed modifications to include the Illumina Nextera adapters and barcodes. Moreover, we have also modified Table 1 where SE and *t*-values were erroneously preceded by a negative sign and are now shown between parentheses. 

The following information is about the primer used, in Section 2.3. and now reads:

“B969F (5′-AATGATACGGCGACCACCGAGATCTACAC-NNNNNNNN-TCGTCGGCAGCGTCAGATGTGTATAAGAGACAG**ACGCGHNRAACCTTACC**-3′) and BA1406R (5′-CAAGCAGAAGACGGCATACGAGAT-NNNNNNNN-GTCTCGTGGGCTCGGAGATGTGTATAAGAGACAG**ACGGGCRGTGWGTRCAA**-3′), where the Nextera adaptors (L and R arm) are in normal font (to either side of the barcodes), the barcodes are represented by NNNNNNNN, and the specific primer regions are bold + underlined.”

The following table is the correct one: 

The changes do not affect the scientific results. The manuscript will be updated and the original will remain online on the article webpage, with a reference to this Correction.

## Figures and Tables

**Table 1:** Comparisons of gut microbiome of magpie and great spotted cuckoo nestlings and of great spotted cuckoo adults in terms of bacterial richness, α-diversity, composition (weighed and unweighed unifrac β-diversity) and abundance and prevalence of bacterial families.

VARIABLE CONSIDERED	TYPE OF SAMPLE			COMPARISONS				
	(A)	(B)	(C)	ANOVA		POST-HOC (Tukey tests)		
	Adult cuckoos	Nestling cuckoos	Nestling magpies					
	(*N* = 6)	(*N* = 12)	(*N* = 7)			A vs. B	A vs. C	B vs. C
	Mean	Mean	Mean	F_2,22_	*p*	*p*	*p*	*p*
	(SE)	(SE)	(SE)	(SE)	(SE)			
Bacterial Richness	73.8	123	52.3	14.42	**0.0001**	**0.008**	**0.405**	**0.0003**
	(12.1)	(8.5)	(11.2)	(0.19)	**(0.000008)**			
α-diversity (Shannon index)	3.92	4.45	2.66	6.50	**0.006**	0.585	0.106	**0.005**
	(0.43)	(0.31)	(0.4)	(0.03)	**(0.00001)**			
				PERMANOVA		POST-HOC (Pair-wise *t*-tests)		
Microbiome composition				Pseudo-F (SE)	*p* (SE)	*p* (*t*-value)	*p* (*t*-value)	*p* (*t*-value)
Weighed unifrac β-diversity				4.35	**0.00022**	0.559	**0.0002**	**0.0001 (2.63)**
				(0.01)	**(0.00004)**	(0.85)	**(2.87)**	
Unweighed unifrac β-diversity				5.37	**0.0001**	**0.0002**	**0.0007**	**0.0001**
				(0.07)	**(0.0001)**	**(1.72)**	**(2.47)**	**(2.61)**
Abundance of Families (Bray−Curtis distance matrices)				5.33	**0.00013**	0.58	**0.0008**	**0.002 (2.94)**
				(0.03)	**(0.00002)**	(0.9)	**(3.00)**	
Prevalence of Families (Jaccard distance matrices)				6.43	**0.0001**	**0.0034**	**0.0006**	**0.0001 (2.86)**
				(0.12)	**(0.00001)**	**(1.62)**	**(2.55)**	

Bold fonts highlight statistically significant results (*p* < 0.05).
