# Supplementary material for: Gut Microbiota of Great Spotted Cuckoo Nestlings is a Mixture of Those of Their Foster Magpie Siblings and of Cuckoo Adults
Source: Genes (Basel). 2018 Jul 27;9(8):381. doi: 10.3390/genes9080381 (PMC6115760; doi:10.3390/genes9080381)
Supplement: Supplementary file 1 [file genes-09-00381-s001.zip › genes-326337-supplementary-final.docx]

Supplementary Material for Gut microbiota of great spotted cuckoo nestlings is a mixture of those of their foster magpie siblings and of cuckoo adults

Magdalena Ruiz-Rodríguez, Manuel Martín-Vivaldi, Manuel Martínez-Bueno
and Juan José Soler ^3,5,^*

**Table S1.** Comparison (Kruskal-Wallis tests (KW), mean (SE) values across 10 rarefactions at 3300 sequences depth) of the abundances of each particular Operational Taxonomic Units (OTU) among the cloacae samples obtained from adult and nestling great-spotted cuckoos as good as nestling magpies. The table shows only the results for the 35 OTUs (out of 741 analyzed) that obtained a *p*-value lesser than 0.01, and both *p* and False Discovery rate (FDR) *p* (correction for multiple tests) are presented (significant *p* < 0.05 for each kind of *p* value are highlighted in bold). The mean (SE) number of sequences for each of the types of samples calculated for the first rarefaction is presented.

| **OTU ID** | **Phylum** | **Family** | **Genus species** | **KW test value**  **Mean (SE)** | ***p* value**  **Mean (SE)** | **FDR *p* value**  **Mean (SE)** | **Adult cuckoos**  **Mean (SE)** | **Nestling cuckoos**  **Mean (SE)** | **Nestling magpies**  **Mean (SE)** |
| --- | --- | --- | --- | --- | --- | --- | --- | --- | --- |
| OTU308 | Firmicutes | Lachnospiraceae | *[Ruminococcus]* | 18.70 (0.00) | 0.0001 (0.0000) | 0.0225 (0.0020) | 67.33 (47.56) | 0.00 (0.00) | 0.00 (0.00) |
| OTU546 | Actinobacteria | Coriobacteriaceae | *Eggerthella lenta* | 16.63 (0.61) | 0.0004 (0.0001) | 0.0335 (0.0043) | 8.50 (2.98) | 0.42 (0.29) | 0.00 (0.00) |
| - | Proteobacteria | Enterobacteriaceae |  | 15.66 (0.23) | 0.0004 (0.0000) | 0.0361 (0.0012) | 0.33 (0.21) | 5.67 (2.99) | 42.86 (11.07) |
| OTU160 | Proteobacteria | Desulfovibrionaceae | *Bilophila* | 15.41 (0.21) | 0.0005 (0.0000) | 0.0373 (0.0020) | 1.83 (1.83) | 19.83 (6.82) | 0.00 (0.00) |
| OTU540 | Firmicutes |  |  | 16.32 (0.75) | 0.0006 (0.0003) | 0.0388 (0.0068) | 95.67 (45.72) | 0.67 (0.47) | 0.00 (0.00) |
| OTU50 | Firmicutes | Clostridiaceae | *Candidatus Arthromitus* | 17.56 (0.99) | 0.0006 (0.0004) | 0.0355 (0.0084) | 0.00 (0.00) | 0.00 (0.00) | 7.29 (3.52) |
| OTU286 | Bacteroidetes | Porphyromonadaceae | *Parabacteroides* | 15.45 (0.53) | 0.0007 (0.0003) | 0.0405 (0.0045) | 66.67 (35.77) | 11.58 (11.40) | 0.00 (0.00) |
| OTU29 | Proteobacteria | Enterobacteriaceae |  | 14.69 (0.29) | 0.0007 (0.0001) | 0.0419 (0.0043) | 0.00 (0.00) | 1.17 (0.73) | 8.00 (2.93) |
| OTU601 | Firmicutes | Veillonellaceae | *Veillonella* | 14.34 (0.00) | 0.0008 (0.0000) | 0.0402 (0.0017) | 337.17 (276.95) | 0.00 (0.00) | 0.00 (0.00) |
| OTU262 | Firmicutes | Ruminococcaceae |  | 14.90 (0.56) | 0.0008 (0.0002) | 0.0431 (0.0068) | 38.00 (26.39) | 0.17 (0.17) | 0.00 (0.00) |
| OTU16 | Proteobacteria | Enterobacteriaceae |  | 14.12 (0.21) | 0.0009 (0.0001) | 0.0458 (0.0036) | 0.50 (0.34) | 4.92 (2.34) | 42.00 (13.27) |
| OTU292 | Firmicutes | Erysipelotrichaceae | *[Eubacterium]= dolichum* | 13.93 (0.14) | 0.0010 (0.0001) | 0.0453 (0.0024) | 44.17 (14.66) | 10.50 (5.30) | 0.00 (0.00) |
| OTU21 | Firmicutes | Clostridiaceae | *Candidatus Arthromitus* | 14.12 (0.54) | 0.0012 (0.0004) | 0.0526 (0.0084) | 0.00 (0.00) | 0.00 (0.00) | 31.71 (19.98) |
| OTU36 | Proteobacteria | Enterobacteriaceae |  | 13.77 (0.50) | 0.0014 (0.0005) | 0.0523 (0.0075) | 0.00 (0.00) | 1.25 (0.65) | 8.86 (2.75) |
| OTU547 | Firmicutes | Ruminococcaceae | *Ruminococcus* | 14.65 (1.02) | 0.0016 (0.0006) | 0.0524 (0.0115) | 1.33 (0.56) | 0.00 (0.00) | 0.00 (0.00) |
| OTU52 | Proteobacteria | Enterobacteriaceae |  | 13.40 (0.60) | 0.0017 (0.0004) | 0.0647 (0.0095) | 0.00 (0.00) | 1.00 (0.56) | 6.29 (1.02) |
| OTU1 | Proteobacteria | Enterobacteriaceae |  | 12.57 (0.08) | 0.0019 (0.0001) | 0.0759 (0.0039) | 8.33 (4.71) | 151.00 (81.85) | 1148.71 (368.70) |
| OTU6 | Actinobacteria | Corynebacteriaceae | *Corynebacterium* | 14.26 (1.28) | 0.0026 (0.0009) | 0.0672 (0.0159) | 0.00 (0.00) | 0.50 (0.42) | 150.14 (118.45) |
| OTU118 | Proteobacteria | Desulfovibrionaceae | *Bilophila* | 12.05 (0.38) | 0.0027 (0.0003) | 0.0798 (0.0072) | 48.83 (22.18) | 0.08 (0.08) | 0.00 (0.00) |
| OTU20 | Proteobacteria | Enterobacteriaceae |  | 12.17 (0.57) | 0.0032 (0.0009) | 0.0826 (0.0132) | 0.00 (0.00) | 0.00 (0.00) | 29.57 (13.93) |
| OTU7 | Firmicutes | Clostridiaceae | *Clostridium* | 12.38 (0.69) | 0.0034 (0.0014) | 0.0784 (0.0129) | 0.00 (0.00) | 0.00 (0.00) | 148.57 (134.42) |
| OTU595 | Firmicutes | Ruminococcaceae |  | 13.97 (1.18) | 0.0041(0.0027) | 0.0756 (0.0235) | 2.33 (0.88) | 0.00 (0.00) | 0.00 (0.00) |
| OTU68 | Proteobacteria | Enterobacteriaceae |  | 11.52 (0.52) | 0.0041(0.0010) | 0.0963 (0.0148) | 0.00 (0.00) | 0.00 (0.00) | 4.86 (1.78) |
| OTU64 | Proteobacteria | Enterobacteriaceae |  | 11.32 (0.33) | 0.0042(0.0012) | 0.0944 (0.0085) | 0.00 (0.00) | 0.00 (0.00) | 5.86 (3.27) |
| OTU313 | Firmicutes |  |  | 10.78 (0.13) | 0.0047(0.0003) | 0.1084 (0.0056) | 56.33 (28.93) | 6.33 (4.85) | 0.00 (0.00) |
| OTU66 | Firmicutes | Clostridiaceae | *Candidatus Arthromitus* | 11.71 (0.72) | 0.0049(0.0017) | 0.0997 (0.0174) | 0.00 (0.00) | 0.00 (0.00) | 3.86 (2.77) |
| OTU55 | Proteobacteria | Enterobacteriaceae |  | 11.05 (0.48) | 0.0057(0.0021) | 0.1081 (0.0173) | 0.00 (0.00) | 0.00 (0.00) | 6.86 (3.01) |
| OTU569 | Actinobacteria | Bifidobacteriaceae | *Bifidobacterium* | 11.56 (0.84) | 0.0069(0.0034) | 0.1033 (0.0262) | 53.83 (50.50) | 0.00 (0.00) | 0.00 (0.00) |
| OTU220 | Firmicutes | Lachnospiraceae |  | 9.90 (0.22) | 0.0075(0.0009) | 0.1350 (0.0115) | 73.50 (48.07) | 1.50 (1.50) | 0.00 (0.00) |
| OTU352 | Firmicutes | Ruminococcaceae | *Oscillospira* | 10.02 (0.43) | 0.0082(0.0018) | 0.1306 (0.0132) | 0.00 (0.00) | 4.42 (2.50) | 0.00 (0.00) |
| OTU22 | Proteobacteria | Enterobacteriaceae |  | 10.18 (0.65) | 0.0083(0.0014) | 0.1423 (0.0173) | 0.00 (0.00) | 0.00 (0.00) | 28.29 (14.00) |
| OTU579 | Firmicutes | Lachnospiraceae | *Coprococcus* | 9.94 (0.37) | 0.0089(0.0031) | 0.1320 (0.0165) | 15.00 (12.87) | 0.00 (0.00) | 0.00 (0.00) |
| OTU493 | Actinobacteria | Coriobacteriaceae |  | 9.94 (0.37) | 0.0089(0.0031) | 0.1310 (0.0162) | 59.00 (56.43) | 0.00 (0.00) | 0.00 (0.00) |
| OTU74 | Proteobacteria | Enterobacteriaceae |  | 9.77 (0.51) | 0.0097(0.0019) | 0.1473 (0.0167) | 0.00 (0.00) | 0.00 (0.00) | 3.43 (2.08) |
| OTU330 | Firmicutes | Ruminococcaceae |  | 9.56 (0.37) | 0.0098(0.0019) | 0.1531 (0.0151) | 7.00 (3.56) | 5.75 (2.44) | 0.00 (0.00) |

**Table S2.** List of OTUs with a prevalence higher than 25% in any of the three types of samples. The table shows the correspondence between OTUs IDs in Fig.1, taxonomy and labels assigned by QIIME in the picking OTUs procedure.

| **OTU ID.** | **Phylum** | **Class** | **Order** | **Family** | **Genus species** | **Illumina label** |
| --- | --- | --- | --- | --- | --- | --- |
| OTU1 | Proteobacteria | Gammaproteobacteria | Enterobacteriales | Enterobacteriaceae |  | 797229 |
| OTU2 | Proteobacteria | Gammaproteobacteria | Enterobacteriales | Enterobacteriaceae |  | 823118 |
| OTU3 | Proteobacteria | Epsilonproteobacteria | Campylobacterales | Campylobacteraceae | *Campylobacter* | 4361046 |
| OTU4 | Firmicutes | Clostridia | Clostridiales | Dehalobacteriaceae | *Dehalobacterium* | 187409 |
| OTU5 | Firmicutes | Clostridia | Clostridiales | Lachnospiraceae |  | 436032 |
| OTU6 | Actinobacteria | Actinobacteria | Actinomycetales | Corynebacteriaceae | *Corynebacterium* | New.ReferenceOTU152 |
| OTU7 | Firmicutes | Clostridia | Clostridiales | Clostridiaceae | *Clostridium* | 582630 |
| OTU8 | Firmicutes | Bacilli | Lactobacillales | Enterococcaceae | *Enterococcus* | 1085646 |
| OTU9 | Bacteroidetes | Bacteroidia | Bacteroidales | [Odoribacteraceae] | *Butyricimonas* | 187121 |
| OTU10 | Proteobacteria | Gammaproteobacteria | Enterobacteriales | Enterobacteriaceae |  | 4423027 |
| OTU11 | Firmicutes | Clostridia | Clostridiales | Lachnospiraceae | *[Ruminococcus] gnavus* | New.ReferenceOTU150 |
| OTU12 | Firmicutes | Clostridia | Clostridiales | Ruminococcaceae |  | 572461 |
| OTU13 | Bacteroidetes | [Saprospirae] | [Saprospirales] | Chitinophagaceae | *Sediminibacterium* | 2792167 |
| OTU14 | Proteobacteria | Epsilonproteobacteria | Campylobacterales | Campylobacteraceae | *Campylobacter* | 284123 |
| OTU15 | Proteobacteria | Gammaproteobacteria | Enterobacteriales | Enterobacteriaceae |  | 301184 |
| OTU16 | Proteobacteria | Gammaproteobacteria | Enterobacteriales | Enterobacteriaceae |  | 345951 |
| OTU17 | Proteobacteria | Gammaproteobacteria | Enterobacteriales | Enterobacteriaceae |  | 822899 |
| OTU18 | Proteobacteria | Gammaproteobacteria | Enterobacteriales | Enterobacteriaceae |  | 1111874 |
| OTU19 | Proteobacteria | Gammaproteobacteria | Enterobacteriales | Enterobacteriaceae |  | 814952 |
| OTU20 | Proteobacteria | Gammaproteobacteria | Enterobacteriales | Enterobacteriaceae |  | 618010 |
| OTU21 | Firmicutes | Clostridia | Clostridiales | Clostridiaceae | *Candidatus Arthromitus* | 16195 |
| OTU22 | Proteobacteria | Gammaproteobacteria | Enterobacteriales | Enterobacteriaceae |  | 812379 |
| OTU23 | Bacteroidetes | Bacteroidia | Bacteroidales | Porphyromonadaceae | *Parabacteroides* | 2058521 |
| OTU24 | Firmicutes | Clostridia | Clostridiales | Lachnospiraceae |  | 815573 |
| OTU25 | Proteobacteria | Gammaproteobacteria | Enterobacteriales | Enterobacteriaceae |  | 224212 |
| OTU26 | Firmicutes | Erysipelotrichi | Erysipelotrichales | Erysipelotrichaceae |  | 356760 |
| OTU27 | Proteobacteria | Gammaproteobacteria | Enterobacteriales | Enterobacteriaceae |  | 3151924 |
| OTU28 | Proteobacteria | Gammaproteobacteria | Enterobacteriales | Enterobacteriaceae |  | 3825935 |
| OTU29 | Proteobacteria | Gammaproteobacteria | Enterobacteriales | Enterobacteriaceae |  | 4382827 |
| OTU30 | Firmicutes | Clostridia | Clostridiales |  |  | 199336 |
| OTU31 | Proteobacteria | Gammaproteobacteria | Enterobacteriales | Enterobacteriaceae |  | 1791578 |
| OTU32 | Proteobacteria | Gammaproteobacteria | Enterobacteriales | Enterobacteriaceae |  | 819636 |
| OTU33 | Firmicutes | Bacilli | Lactobacillales |  |  | 1111582 |
| OTU34 | Proteobacteria | Gammaproteobacteria | Enterobacteriales | Enterobacteriaceae |  | 1109623 |
| OTU35 | Firmicutes | Clostridia | Clostridiales |  |  | 157193 |
| OTU36 | Proteobacteria | Gammaproteobacteria | Enterobacteriales | Enterobacteriaceae |  | 1109844 |
| OTU37 | Firmicutes | Clostridia | Clostridiales | Lachnospiraceae | *Clostridium piliforme* | New.ReferenceOTU251 |
| OTU38 | Firmicutes | Clostridia | Clostridiales | Ruminococcaceae |  | 332507 |
| OTU39 | Firmicutes | Clostridia | Clostridiales | Ruminococcaceae | *Ruminococcus* | 195415 |
| OTU40 | Proteobacteria | Gammaproteobacteria | Enterobacteriales | Enterobacteriaceae |  | 1110417 |
| OTU41 | Firmicutes | Clostridia | Clostridiales | Lachnospiraceae |  | 2722893 |
| OTU42 | Proteobacteria | Alphaproteobacteria | Caulobacterales | Caulobacteraceae | *Phenylobacterium* | 353051 |
| OTU43 | Firmicutes | Clostridia | Clostridiales | Clostridiaceae | *Clostridium* | 287620 |
| OTU44 | Firmicutes | Clostridia | Clostridiales | Clostridiaceae |  | 338222 |
| OTU45 | Bacteroidetes | Bacteroidia | Bacteroidales | Rikenellaceae |  | 357046 |
| OTU46 | Firmicutes | Clostridia | Clostridiales | Lachnospiraceae |  | 182723 |
| OTU47 | Proteobacteria | Gammaproteobacteria | Enterobacteriales | Enterobacteriaceae |  | 635304 |
| OTU48 | Bacteroidetes | Bacteroidia | Bacteroidales | Bacteroidaceae | *Bacteroides* | 772282 |
| OTU49 | Firmicutes | Bacilli | Lactobacillales | Enterococcaceae | *Enterococcus* | 588755 |
| OTU50 | Firmicutes | Clostridia | Clostridiales | Clostridiaceae | *Candidatus Arthromitus* | 1058952 |
| OTU51 | Firmicutes | Clostridia | Clostridiales | Ruminococcaceae | *Ruminococcus* | 1828413 |
| OTU52 | Proteobacteria | Gammaproteobacteria | Enterobacteriales | Enterobacteriaceae |  | 1109362 |
| OTU53 | Firmicutes | Clostridia | Clostridiales | Clostridiaceae |  | 4482110 |
| OTU54 | Actinobacteria | Actinobacteria | Actinomycetales | Propionibacteriaceae | *Propionibacterium acnes* | 1098473 |
| OTU55 | Proteobacteria | Gammaproteobacteria | Enterobacteriales | Enterobacteriaceae |  | 222043 |
| OTU56 | Proteobacteria | Gammaproteobacteria | Aeromonadales | Aeromonadaceae |  | 834097 |
| OTU57 | Proteobacteria | Gammaproteobacteria | Aeromonadales | Aeromonadaceae |  | 837030 |
| OTU58 | Actinobacteria | Coriobacteriia | Coriobacteriales | Coriobacteriaceae |  | 338145 |
| OTU59 | Actinobacteria | Coriobacteriia | Coriobacteriales | Coriobacteriaceae |  | 2127939 |
| OTU60 | Proteobacteria | Gammaproteobacteria | Pseudomonadales | Pseudomonadaceae | *Pseudomonas veronii* | 930834 |
| OTU61 | Firmicutes | Bacilli | Lactobacillales |  |  | 543824 |
| OTU62 | Firmicutes | Clostridia | Clostridiales | Clostridiaceae |  | 314402 |
| OTU63 | Proteobacteria | Gammaproteobacteria | Enterobacteriales | Enterobacteriaceae | *Klebsiella* | 808486 |
| OTU64 | Proteobacteria | Gammaproteobacteria | Enterobacteriales | Enterobacteriaceae |  | New.ReferenceOTU76 |
| OTU65 | Actinobacteria | Coriobacteriia | Coriobacteriales | Coriobacteriaceae | *Atopobium* | New.ReferenceOTU132 |
| OTU66 | Firmicutes | Clostridia | Clostridiales | Clostridiaceae | *Candidatus Arthromitus* | 181348 |
| OTU67 | Bacteroidetes | Bacteroidia | Bacteroidales | Bacteroidaceae | *Bacteroides fragilis* | 2200896 |
| OTU68 | Proteobacteria | Gammaproteobacteria | Enterobacteriales | Enterobacteriaceae |  | 562618 |
| OTU69 | Firmicutes | Erysipelotrichi | Erysipelotrichales | Erysipelotrichaceae | *[Eubacterium] dolichum* | 1904686 |
| OTU70 | Firmicutes | Clostridia | Clostridiales | Lachnospiraceae |  | 3645685 |
| OTU71 | Actinobacteria | Actinobacteria | Actinomycetales | Corynebacteriaceae | *Corynebacterium* | 1051939 |
| OTU72 | Actinobacteria | Coriobacteriia | Coriobacteriales | Coriobacteriaceae |  | 1141218 |
| OTU73 | Firmicutes | Clostridia | Clostridiales | Peptostreptococcaceae |  | 534957 |
| OTU74 | Proteobacteria | Gammaproteobacteria | Enterobacteriales | Enterobacteriaceae |  | 300729 |
| OTU75 | Firmicutes | Clostridia | Clostridiales | Ruminococcaceae |  | 317286 |
| OTU76 | Proteobacteria | Gammaproteobacteria | Enterobacteriales | Enterobacteriaceae |  | 1110402 |
| OTU77 | Firmicutes | Clostridia | Clostridiales | Clostridiaceae |  | 1147925 |
| OTU78 | Proteobacteria | Epsilonproteobacteria | Campylobacterales | Campylobacteraceae | *Campylobacter* | New.ReferenceOTU136 |
| OTU79 | Proteobacteria | Epsilonproteobacteria | Campylobacterales | Campylobacteraceae | *Campylobacter* | New.ReferenceOTU214 |
| OTU80 | Firmicutes | Clostridia | Clostridiales | [Tissierellaceae] | *Gallicola* | 768514 |
| OTU81 | Firmicutes | Bacilli | Bacillales | Staphylococcaceae | *Staphylococcus* | 1101177 |
| OTU82 | Firmicutes | Erysipelotrichi | Erysipelotrichales | Erysipelotrichaceae |  | 4390365 |
| OTU83 | Proteobacteria | Epsilonproteobacteria | Campylobacterales | Campylobacteraceae | *Campylobacter* | New.ReferenceOTU162 |
| OTU84 | Proteobacteria | Alphaproteobacteria | Rhizobiales | Methylobacteriaceae | *Methylobacterium adhaesivum* | 895733 |
| OTU85 | Bacteroidetes | Bacteroidia | Bacteroidales | Bacteroidaceae | *Bacteroides* | 2656868 |
| OTU86 | Firmicutes | Clostridia | Clostridiales | Peptostreptococcaceae |  | 308309 |
| OTU87 | Proteobacteria | Alphaproteobacteria | Sphingomonadales | Sphingomonadaceae | *Kaistobacter* | 949062 |
| OTU88 | Bacteroidetes | Bacteroidia | Bacteroidales | Bacteroidaceae | *Bacteroides* | 1135084 |
| OTU89 | Proteobacteria | Deltaproteobacteria | Desulfovibrionales | Desulfovibrionaceae | *Desulfovibrio* | 4453773 |
| OTU90 | Actinobacteria | Actinobacteria | Actinomycetales | Micrococcaceae |  | 824723 |
| OTU91 | Fusobacteria | Fusobacteriia | Fusobacteriales | Fusobacteriaceae | *Fusobacterium* | 1654477 |
| OTU92 | Proteobacteria | Alphaproteobacteria | Caulobacterales | Caulobacteraceae |  | 998905 |
| OTU93 | Proteobacteria | Gammaproteobacteria | Enterobacteriales | Enterobacteriaceae | *Erwinia* | 441019 |
| OTU94 | Firmicutes | Bacilli | Lactobacillales | Streptococcaceae | *Lactococcus* | 593781 |
| OTU95 | Bacteroidetes | Bacteroidia | Bacteroidales | Bacteroidaceae | *Bacteroides* | New.ReferenceOTU131 |
| OTU96 | Actinobacteria | Actinobacteria | Actinomycetales | Geodermatophilaceae |  | 975341 |
| OTU97 | Bacteroidetes | Bacteroidia | Bacteroidales | [Paraprevotellaceae] | *Paraprevotella* | 193534 |
| OTU98 | Firmicutes | Clostridia | Clostridiales | Lachnospiraceae | *Blautia producta* | New.ReferenceOTU75 |
| OTU99 | Firmicutes | Clostridia | Clostridiales |  |  | 582616 |
| OTU100 | Proteobacteria | Gammaproteobacteria | Enterobacteriales | Enterobacteriaceae |  | New.ReferenceOTU166 |
| OTU101 | Bacteroidetes | Bacteroidia | Bacteroidales | Porphyromonadaceae | *Parabacteroides* | 4372003 |
| OTU102 | Firmicutes | Clostridia | Clostridiales | Clostridiaceae | *Clostridium* | New.ReferenceOTU155 |
| OTU103 | Proteobacteria | Alphaproteobacteria | Rhizobiales | Methylobacteriaceae | *Methylobacterium* | 68458 |
| OTU104 | Firmicutes | Clostridia | Clostridiales |  |  | 336627 |
| OTU105 | Firmicutes | Clostridia | Clostridiales | Clostridiaceae |  | New.ReferenceOTU231 |
| OTU106 | Proteobacteria | Gammaproteobacteria | Enterobacteriales | Enterobacteriaceae |  | New.ReferenceOTU165 |
| OTU107 | Actinobacteria | Actinobacteria | Actinomycetales | Nocardioidaceae |  | 973424 |
| OTU108 | Firmicutes | Bacilli | Lactobacillales | Streptococcaceae | *Lactococcus* | 335256 |
| OTU109 | Firmicutes | Clostridia | Clostridiales | Clostridiaceae |  | 345448 |
| OTU110 | Firmicutes | Clostridia | Clostridiales | Ruminococcaceae | *Oscillospira* | 179215 |
| OTU111 | Firmicutes | Bacilli | Bacillales | Bacillaceae | *Bacillus muralis* | 854050 |
| OTU112 | Firmicutes | Clostridia | Clostridiales | Clostridiaceae |  | 318645 |
| OTU113 | Actinobacteria | Actinobacteria | Actinomycetales | Micrococcaceae |  | 929901 |
| OTU114 | Proteobacteria | Gammaproteobacteria | Enterobacteriales | Enterobacteriaceae |  | 588216 |
| OTU115 | Proteobacteria | Gammaproteobacteria | Enterobacteriales | Enterobacteriaceae |  | 801438 |
| OTU116 | Bacteroidetes | Flavobacteriia | Flavobacteriales | [Weeksellaceae] | *Chryseobacterium* | 544076 |
| OTU117 | Proteobacteria | Gammaproteobacteria | Enterobacteriales | Enterobacteriaceae |  | 694616 |
| OTU118 | Proteobacteria | Deltaproteobacteria | Desulfovibrionales | Desulfovibrionaceae | *Bilophila* | New.ReferenceOTU215 |
| OTU119 | Firmicutes | Clostridia | Clostridiales | Ruminococcaceae |  | 183932 |
| OTU120 | Proteobacteria | Betaproteobacteria | Burkholderiales | Alcaligenaceae |  | New.ReferenceOTU278 |
| OTU121 | Firmicutes | Bacilli | Lactobacillales | Enterococcaceae | *Enterococcus* | 1008941 |
| OTU122 | Bacteroidetes | Bacteroidia | Bacteroidales | Bacteroidaceae | *Bacteroides* | 354850 |
| OTU123 | Firmicutes | Clostridia | Clostridiales | Clostridiaceae |  | 347451 |
| OTU124 | Bacteroidetes | Bacteroidia | Bacteroidales | Porphyromonadaceae | *Parabacteroides distasonis* | 577294 |
| OTU125 | Proteobacteria | Alphaproteobacteria | Rhizobiales | Methylobacteriaceae | *Methylobacterium* | 4323871 |
| OTU126 | Firmicutes | Clostridia | Clostridiales | Clostridiaceae |  | 3171053 |
| OTU127 | Fusobacteria | Fusobacteriia | Fusobacteriales | Fusobacteriaceae | *Fusobacterium* | 298592 |
| OTU128 | Proteobacteria | Gammaproteobacteria | Enterobacteriales | Enterobacteriaceae |  | 168313 |
| OTU129 | Bacteroidetes | Bacteroidia | Bacteroidales | Bacteroidaceae | *Bacteroides fragilis* | 1776713 |
| OTU130 | Actinobacteria | Actinobacteria | Actinomycetales | Geodermatophilaceae |  | 818388 |
| OTU131 | Firmicutes | Clostridia | Clostridiales | Ruminococcaceae |  | 185391 |
| OTU132 | Firmicutes | Clostridia | Clostridiales | Lachnospiraceae |  | 2254001 |
| OTU133 | Firmicutes | Bacilli | Bacillales | Bacillaceae |  | 347529 |
| OTU134 | Bacteroidetes | Bacteroidia | Bacteroidales | Bacteroidaceae | *Bacteroides* | 4425495 |
| OTU135 | Firmicutes | Bacilli | Lactobacillales | Enterococcaceae | *Enterococcus* | 839152 |
| OTU136 | Firmicutes | Bacilli | Lactobacillales | Enterococcaceae | *Enterococcus* | 4469032 |
| OTU137 | Bacteroidetes | Bacteroidia | Bacteroidales | Bacteroidaceae | *Bacteroides fragilis* | 308598 |
| OTU138 | Actinobacteria | Actinobacteria | Actinomycetales | Corynebacteriaceae | *Corynebacterium* | 1080820 |
| OTU139 | Actinobacteria | Actinobacteria | Actinomycetales | Propionibacteriaceae | *Propionibacterium acnes* | 370772 |
| OTU140 | Firmicutes | Clostridia | Clostridiales | Lachnospiraceae |  | New.ReferenceOTU254 |
| OTU141 | Firmicutes | Clostridia | Clostridiales | Lachnospiraceae | *Coprococcus* | New.ReferenceOTU52 |
| OTU142 | Firmicutes | Clostridia | Clostridiales | Lachnospiraceae |  | New.ReferenceOTU167 |
| OTU143 | Bacteroidetes | Bacteroidia | Bacteroidales | Bacteroidaceae | *Bacteroides* | 305946 |
| OTU144 | Firmicutes | Clostridia | Clostridiales | Ruminococcaceae |  | 518444 |
| OTU145 | Firmicutes | Clostridia | Clostridiales |  |  | 224379 |
| OTU146 | Actinobacteria | Actinobacteria | Actinomycetales | Microbacteriaceae | *Microbacterium* | 459107 |
| OTU147 | Firmicutes | Clostridia | Clostridiales | Ruminococcaceae |  | 572751 |
| OTU148 | Proteobacteria | Gammaproteobacteria | Enterobacteriales | Enterobacteriaceae | *Proteus* | 4484484 |
| OTU149 | Firmicutes | Bacilli | Turicibacterales | Turicibacteraceae | *Turicibacter* | 101343 |
| OTU150 | Firmicutes | Clostridia | Clostridiales | Lachnospiraceae |  | 366797 |
| OTU151 | Firmicutes | Clostridia | Clostridiales | Lachnospiraceae | *[Ruminococcus]* | New.ReferenceOTU139 |
| OTU152 | Firmicutes | Bacilli | Bacillales | Staphylococcaceae | *Staphylococcus* | 1075891 |
| OTU153 | Firmicutes | Bacilli | Lactobacillales | Enterococcaceae | *Enterococcus* | 539447 |
| OTU154 | Proteobacteria | Alphaproteobacteria | Rhizobiales | Methylobacteriaceae | *Methylobacterium* | 335000 |
| OTU155 | Actinobacteria | Actinobacteria | Actinomycetales | Propionibacteriaceae | *Propionibacterium acnes* | 1087597 |
| OTU156 | Firmicutes | Bacilli | Lactobacillales | Enterococcaceae | *Enterococcus* | 295146 |
| OTU157 | Firmicutes | Clostridia | Clostridiales | Clostridiaceae |  | 346666 |
| OTU158 | Bacteroidetes | Bacteroidia | Bacteroidales | Bacteroidaceae | *Bacteroides fragilis* | New.ReferenceOTU234 |
| OTU159 | Proteobacteria | Gammaproteobacteria | Enterobacteriales | Enterobacteriaceae |  | 3441309 |
| OTU160 | Proteobacteria | Deltaproteobacteria | Desulfovibrionales | Desulfovibrionaceae | *Bilophila* | 359872 |
| OTU161 | Firmicutes | Clostridia | Clostridiales |  |  | 198928 |
| OTU162 | Firmicutes | Clostridia | Clostridiales | Ruminococcaceae |  | 559302 |
| OTU163 | Firmicutes | Clostridia | Clostridiales | Ruminococcaceae |  | 339121 |
| OTU164 | Firmicutes | Clostridia | Clostridiales | Clostridiaceae | *Clostridium* | 555688 |
| OTU165 | Firmicutes | Clostridia | Clostridiales | Ruminococcaceae |  | New.ReferenceOTU95 |
| OTU166 | Firmicutes | Clostridia | Clostridiales | Lachnospiraceae |  | 548503 |
| OTU167 | Firmicutes | Bacilli | Lactobacillales | Enterococcaceae | *Enterococcus* | 513646 |
| OTU168 | Firmicutes | Clostridia | Clostridiales | Lachnospiraceae | *[Ruminococcus]* | 3421266 |
| OTU169 | Bacteroidetes | Bacteroidia | Bacteroidales | Bacteroidaceae | *Bacteroides* | 568118 |
| OTU170 | Bacteroidetes | Bacteroidia | Bacteroidales | [Odoribacteraceae] | *Odoribacter* | 208843 |
| OTU171 | Bacteroidetes | Bacteroidia | Bacteroidales | Bacteroidaceae | *Bacteroides* | 4340358 |
| OTU172 | Firmicutes | Clostridia | Clostridiales | Lachnospiraceae | *Coprococcus* | 358834 |
| OTU173 | Firmicutes | Erysipelotrichi | Erysipelotrichales | Erysipelotrichaceae | *[Eubacterium] dolichum* | 579851 |
| OTU174 | Bacteroidetes | Bacteroidia | Bacteroidales |  |  | 344154 |
| OTU175 | Actinobacteria | Actinobacteria | Actinomycetales | Corynebacteriaceae | *Corynebacterium* | 1039051 |
| OTU176 | Tenericutes | Mollicutes | RF39 |  |  | 513605 |
| OTU177 | Firmicutes | Clostridia | Clostridiales | Ruminococcaceae |  | 580383 |
| OTU178 | Fusobacteria | Fusobacteriia | Fusobacteriales | Fusobacteriaceae | *Fusobacterium* | 1873204 |
| OTU179 | Firmicutes | Clostridia | Clostridiales | Lachnospiraceae |  | New.ReferenceOTU279 |
| OTU180 | Firmicutes | Clostridia | Clostridiales | Lachnospiraceae |  | New.ReferenceOTU15 |
| OTU181 | Firmicutes | Clostridia | Clostridiales | Ruminococcaceae |  | 185712 |
| OTU182 | Actinobacteria | Actinobacteria | Actinomycetales | Corynebacteriaceae | *Corynebacterium kroppenstedtii* | 444738 |
| OTU183 | Firmicutes | Clostridia | Clostridiales | Clostridiaceae |  | 317288 |
| OTU184 | Bacteroidetes | Bacteroidia | Bacteroidales | Rikenellaceae |  | 953855 |
| OTU185 | Firmicutes | Clostridia | Clostridiales | Lachnospiraceae | *[Ruminococcus]* | 563086 |
| OTU186 | Firmicutes | Bacilli | Bacillales | Bacillaceae |  | 1028036 |
| OTU187 | Firmicutes | Clostridia | Clostridiales | Ruminococcaceae | *Ruminococcus* | New.ReferenceOTU274 |
| OTU188 | Firmicutes | Clostridia | Clostridiales | Lachnospiraceae | *Dorea* | New.ReferenceOTU90 |
| OTU189 | Bacteroidetes | Bacteroidia | Bacteroidales | [Barnesiellaceae] |  | 846798 |
| OTU190 | Bacteroidetes | Bacteroidia | Bacteroidales | Bacteroidaceae | *Bacteroides* | 349809 |
| OTU191 | Firmicutes | Clostridia | Clostridiales |  |  | 192302 |
| OTU192 | Firmicutes | Clostridia | Clostridiales | Ruminococcaceae | *Oscillospira* | 212686 |
| OTU193 | Proteobacteria | Deltaproteobacteria | Desulfovibrionales | Desulfovibrionaceae | *Desulfovibrio* | 308451 |
| OTU194 | Fusobacteria | Fusobacteriia | Fusobacteriales | Fusobacteriaceae | *Fusobacterium* | 2841106 |
| OTU195 | Actinobacteria | Actinobacteria | Actinomycetales |  |  | 4414230 |
| OTU196 | Proteobacteria | Gammaproteobacteria | Enterobacteriales | Enterobacteriaceae | *Trabulsiella* | 546384 |
| OTU197 | Firmicutes | Clostridia | Clostridiales | Ruminococcaceae |  | 166689 |
| OTU198 | Firmicutes | Clostridia | Clostridiales |  |  | 178117 |
| OTU199 | Fusobacteria | Fusobacteriia | Fusobacteriales | Fusobacteriaceae | *Fusobacterium* | 351979 |
| OTU200 | Bacteroidetes | Bacteroidia | Bacteroidales | Porphyromonadaceae | *Parabacteroides distasonis* | 291090 |
| OTU201 | Bacteroidetes | Bacteroidia | Bacteroidales |  |  | 189960 |
| OTU202 | Proteobacteria | Alphaproteobacteria | Rhizobiales | Methylobacteriaceae | *Methylobacterium* | 861160 |
| OTU203 | Bacteroidetes | Bacteroidia | Bacteroidales | Bacteroidaceae | *Bacteroides* | 197537 |
| OTU204 | Firmicutes | Clostridia | Clostridiales | Ruminococcaceae | *Faecalibacterium prausnitzii* | 157297 |
| OTU205 | Firmicutes | Erysipelotrichi | Erysipelotrichales | Erysipelotrichaceae | *Coprobacillus* | 231985 |
| OTU206 | Firmicutes | Clostridia | Clostridiales | Lachnospiraceae |  | New.ReferenceOTU78 |
| OTU207 | Firmicutes | Clostridia | Clostridiales | Ruminococcaceae | *Oscillospira* | 352014 |
| OTU208 | Firmicutes | Clostridia | Clostridiales |  |  | 193906 |
| OTU209 | Bacteroidetes | Bacteroidia | Bacteroidales | Rikenellaceae |  | 112720 |
| OTU210 | Firmicutes | Clostridia | Clostridiales | Ruminococcaceae |  | 2066056 |
| OTU211 | Bacteroidetes | Bacteroidia | Bacteroidales | Rikenellaceae |  | 203209 |
| OTU212 | Proteobacteria | Deltaproteobacteria | Desulfovibrionales | Desulfovibrionaceae | *Desulfovibrio* | 364203 |
| OTU214 | Firmicutes | Clostridia | Clostridiales | Lachnospiraceae | *[Ruminococcus]* | New.ReferenceOTU314 |
| OTU215 | Proteobacteria | Deltaproteobacteria | Desulfovibrionales | Desulfovibrionaceae | *Desulfovibrio* | 510683 |
| OTU216 | Bacteroidetes | Bacteroidia | Bacteroidales | Porphyromonadaceae | *Parabacteroides* | New.ReferenceOTU240 |
| OTU217 | Firmicutes | Clostridia | Clostridiales |  |  | 519763 |
| OTU218 | Firmicutes | Clostridia | Clostridiales | Lachnospiraceae | *[Ruminococcus]* | New.ReferenceOTU315 |
| OTU219 | Firmicutes | Clostridia | Clostridiales | Ruminococcaceae |  | 369827 |
| OTU220 | Firmicutes | Clostridia | Clostridiales | Lachnospiraceae |  | New.ReferenceOTU14 |
| OTU221 | Bacteroidetes | Bacteroidia | Bacteroidales | Bacteroidaceae | *Bacteroides* | 323231 |
| OTU222 | Firmicutes | Clostridia | Clostridiales | Lachnospiraceae | *[Ruminococcus]* | New.ReferenceOTU239 |
| OTU223 | Firmicutes | Clostridia | Clostridiales | Ruminococcaceae |  | 575844 |
| OTU224 | Bacteroidetes | Bacteroidia | Bacteroidales | Bacteroidaceae | *Bacteroides* | New.ReferenceOTU43 |
| OTU225 | Firmicutes | Clostridia | Clostridiales f__ |  |  | 287786 |
| OTU226 | Proteobacteria | Gammaproteobacteria | Aeromonadales | Succinivibrionaceae |  | New.ReferenceOTU126 |
| OTU227 | Firmicutes | Erysipelotrichi | Erysipelotrichales | Erysipelotrichaceae | *[Eubacterium]* | New.ReferenceOTU322 |
| OTU228 | Firmicutes | Clostridia | Clostridiales | Ruminococcaceae | *Ruminococcus* | 532187 |
| OTU229 | Firmicutes | Clostridia | Clostridiales | Lachnospiraceae | *Blautia* | New.ReferenceOTU42 |
| OTU230 | Firmicutes | Clostridia | Clostridiales | Ruminococcaceae |  | 198248 |
| OTU231 | Proteobacteria | Alphaproteobacteria | RF32 |  |  | 4435655 |
| OTU232 | Bacteroidetes | Bacteroidia | Bacteroidales | [Barnesiellaceae] |  | 183395 |
| OTU233 | Bacteroidetes | Bacteroidia | Bacteroidales | [Odoribacteraceae] | *Odoribacter* | New.ReferenceOTU317 |
| OTU234 | Firmicutes | Clostridia | Clostridiales | Ruminococcaceae |  | 401384 |
| OTU235 | Firmicutes | Clostridia | Clostridiales | Lachnospiraceae |  | New.ReferenceOTU257 |
| OTU236 | Firmicutes | Clostridia | Clostridiales | Lachnospiraceae | *[Ruminococcus]* | New.ReferenceOTU247 |
| OTU237 | Firmicutes | Clostridia | Clostridiales | Ruminococcaceae |  | 4366089 |
| OTU238 | Bacteroidetes | Bacteroidia | Bacteroidales | Bacteroidaceae | *Bacteroides* | New.ReferenceOTU320 |
| OTU239 | Firmicutes | Clostridia | Clostridiales | Ruminococcaceae |  | New.ReferenceOTU232 |
| OTU240 | Bacteroidetes | Bacteroidia | Bacteroidales | Porphyromonadaceae | *Parabacteroides* | New.ReferenceOTU298 |
| OTU241 | Proteobacteria | Gammaproteobacteria | Enterobacteriales | Enterobacteriaceae |  | 60345 |
| OTU242 | Bacteroidetes | Bacteroidia | Bacteroidales | [Odoribacteraceae] | *Butyricimonas* | 112842 |
| OTU243 | Firmicutes | Clostridia | Clostridiales | Veillonellaceae | *Phascolarctobacterium* | 528753 |
| OTU244 | Firmicutes | Clostridia | Clostridiales | Ruminococcaceae |  | 470117 |
| OTU245 | Firmicutes | Clostridia | Clostridiales | Ruminococcaceae | *Oscillospira* | 581933 |
| OTU246 | Tenericutes | Mollicutes | RF39 |  |  | 235065 |
| OTU247 | Proteobacteria | Betaproteobacteria | Burkholderiales | Alcaligenaceae | *Sutterella* | 1105376 |
| OTU248 | Firmicutes | Clostridia | Clostridiales | Ruminococcaceae | *Oscillospira* | New.ReferenceOTU230 |
| OTU249 | Firmicutes | Clostridia | Clostridiales | Lachnospiraceae | *[Ruminococcus] gnavus* | New.ReferenceOTU267 |
| OTU250 | Bacteroidetes | Bacteroidia | Bacteroidales | [Paraprevotellaceae] | *Paraprevotella* | 366147 |
| OTU251 | Bacteroidetes | Bacteroidia | Bacteroidales | Bacteroidaceae | *Bacteroides* | 3325758 |
| OTU252 | Firmicutes | Clostridia | Clostridiales | Lachnospiraceae |  | New.ReferenceOTU299 |
| OTU253 | Proteobacteria | Gammaproteobacteria | Legionellales | Coxiellaceae | *Rickettsiella* | 678291 |
| OTU254 | Proteobacteria | Alphaproteobacteria | RF32 |  |  | 178491 |
| OTU255 | Firmicutes | Clostridia | Clostridiales |  |  | New.ReferenceOTU145 |
| OTU256 | Firmicutes | Clostridia | Clostridiales | Lachnospiraceae |  | 4333499 |
| OTU257 | Bacteroidetes | Bacteroidia | Bacteroidales | Rikenellaceae |  | 243150 |
| OTU258 | Firmicutes | Clostridia | Clostridiales | Lachnospiraceae |  | 185059 |
| OTU259 | Firmicutes | Clostridia | Clostridiales |  |  | New.ReferenceOTU213 |
| OTU260 | Firmicutes | Bacilli | Lactobacillales | Streptococcaceae | *Streptococcus* | 1098340 |
| OTU261 | Bacteroidetes | Bacteroidia | Bacteroidales | Porphyromonadaceae | *Parabacteroides* | 851323 |
| OTU262 | Firmicutes | Clostridia | Clostridiales | Ruminococcaceae |  | New.ReferenceOTU54 |
| OTU263 | Bacteroidetes | Bacteroidia | Bacteroidales | Bacteroidaceae | *Bacteroides* | 848315 |
| OTU264 | Bacteroidetes | Bacteroidia | Bacteroidales | Porphyromonadaceae | *Parabacteroides* | New.ReferenceOTU84 |
| OTU265 | Bacteroidetes | Bacteroidia | Bacteroidales | Rikenellaceae |  | 107044 |
| OTU266 | Firmicutes | Clostridia | Clostridiales | Lachnospiraceae |  | 296302 |
| OTU267 | Bacteroidetes | Bacteroidia | Bacteroidales | Bacteroidaceae | *Bacteroides ovatus* | 583656 |
| OTU268 | Bacteroidetes | Bacteroidia | Bacteroidales | Rikenellaceae |  | 1530340 |
| OTU269 | Firmicutes | Clostridia | Clostridiales |  |  | New.ReferenceOTU10 |
| OTU270 | Bacteroidetes | Bacteroidia | Bacteroidales | Rikenellaceae | *Rikenella* | 2529160 |
| OTU271 | Firmicutes | Clostridia | Clostridiales |  |  | New.ReferenceOTU83 |
| OTU272 | Firmicutes | Clostridia | Clostridiales |  |  | New.ReferenceOTU143 |
| OTU273 | Bacteroidetes | Bacteroidia | Bacteroidales | Bacteroidaceae | *Bacteroides* | 336559 |
| OTU274 | Bacteroidetes | Bacteroidia | Bacteroidales | Rikenellaceae |  | 4127460 |
| OTU275 | Bacteroidetes | Bacteroidia | Bacteroidales | Rikenellaceae |  | 157573 |
| OTU276 | Spirochaetes | Spirochaetes | Sphaerochaetales | Sphaerochaetaceae | *Sphaerochaeta* | New.ReferenceOTU37 |
| OTU277 | Cyanobacteria | 4C0d-2 | YS2 |  |  | New.ReferenceOTU121 |
| OTU278 | Proteobacteria | Deltaproteobacteria | Desulfovibrionales | Desulfovibrionaceae | *Bilophila* | 730437 |
| OTU279 | Bacteroidetes | Bacteroidia | Bacteroidales | Rikenellaceae |  | 4336943 |
| OTU280 | Bacteroidetes | Bacteroidia | Bacteroidales | Bacteroidaceae | *Bacteroides* | 364179 |
| OTU281 | Proteobacteria | Alphaproteobacteria | RF32 |  |  | 325850 |
| OTU282 | Firmicutes | Clostridia | Clostridiales | Lachnospiraceae | *[Ruminococcus]* | New.ReferenceOTU22 |
| OTU283 | Firmicutes | Clostridia | Clostridiales | Lachnospiraceae | *[Ruminococcus]* | New.ReferenceOTU289 |
| OTU284 | Bacteroidetes | Bacteroidia | Bacteroidales | Bacteroidaceae | *Bacteroides* | 841290 |
| OTU285 | Firmicutes | Clostridia | Clostridiales | Lachnospiraceae |  | 186888 |
| OTU286 | Bacteroidetes | Bacteroidia | Bacteroidales | Porphyromonadaceae | *Parabacteroides* | 197517 |
| OTU287 | Bacteroidetes | Bacteroidia | Bacteroidales | Bacteroidaceae | *Bacteroides* | 359538 |
| OTU288 | Actinobacteria | Coriobacteriia | Coriobacteriales | Coriobacteriaceae |  | New.ReferenceOTU256 |
| OTU289 | Firmicutes | Clostridia | Clostridiales | Ruminococcaceae | *Oscillospira* | 1110378 |
| OTU290 | Firmicutes | Clostridia | Clostridiales | Ruminococcaceae |  | New.ReferenceOTU242 |
| OTU291 | Bacteroidetes | Bacteroidia | Bacteroidales | Porphyromonadaceae | *Parabacteroides* | New.ReferenceOTU19 |
| OTU292 | Firmicutes | Erysipelotrichi | Erysipelotrichales | Erysipelotrichaceae | *[Eubacterium] dolichum* | New.ReferenceOTU120 |
| OTU293 | Firmicutes | Clostridia | Clostridiales |  |  | 198245 |
| OTU294 | Bacteroidetes | Bacteroidia | Bacteroidales | Bacteroidaceae | *Bacteroides* | New.ReferenceOTU269 |
| OTU295 | Bacteroidetes | Bacteroidia | Bacteroidales | Bacteroidaceae | *Bacteroides* | 3940440 |
| OTU296 | Bacteroidetes | Bacteroidia | Bacteroidales | [Odoribacteraceae] | *Odoribacter* | 73753 |
| OTU297 | Firmicutes | Clostridia | Clostridiales | Ruminococcaceae |  | New.ReferenceOTU40 |
| OTU298 | Lentisphaerae | [Lentisphaeria] | Victivallales | Victivallaceae |  | 4329575 |
| OTU299 | Proteobacteria | Betaproteobacteria | Burkholderiales | Alcaligenaceae | *Sutterella* | 359809 |
| OTU300 | Firmicutes | Clostridia | Clostridiales |  |  | 548021 |
| OTU301 | Firmicutes | Clostridia | Clostridiales | Lachnospiraceae | *Coprococcus* | 3141342 |
| OTU302 | Firmicutes | Clostridia | Clostridiales | Peptostreptococcaceae | *Peptostreptococcus* | 837640 |
| OTU303 | Firmicutes | Clostridia | Clostridiales | Lachnospiraceae |  | New.ReferenceOTU193 |
| OTU304 | Firmicutes | Clostridia | Clostridiales | Ruminococcaceae | *Faecalibacterium prausnitzii* | New.ReferenceOTU13 |
| OTU305 | Bacteroidetes | Bacteroidia | Bacteroidales | [Barnesiellaceae] |  | New.ReferenceOTU241 |
| OTU306 | Firmicutes | Clostridia | Clostridiales | Lachnospiraceae | *Blautia producta* | 158211 |
| OTU307 | Firmicutes | Clostridia | Clostridiales |  |  | New.ReferenceOTU146 |
| OTU308 | Firmicutes | Clostridia | Clostridiales | Lachnospiraceae | *[Ruminococcus]* | New.ReferenceOTU80 |
| OTU309 | Bacteroidetes | Bacteroidia | Bacteroidales | Bacteroidaceae | *Bacteroides ovatus* | 535375 |
| OTU310 | Firmicutes | Clostridia | Clostridiales | Ruminococcaceae |  | 580521 |
| OTU311 | Firmicutes | Clostridia | Clostridiales | Lachnospiraceae |  | 727507 |
| OTU312 | Firmicutes | Clostridia | Clostridiales | Ruminococcaceae |  | 323115 |
| OTU313 | Firmicutes | Clostridia | Clostridiales |  |  | New.ReferenceOTU265 |
| OTU314 | Cyanobacteria | 4C0d-2 | YS2 |  |  | 269386 |
| OTU315 | Proteobacteria | Deltaproteobacteria | Desulfovibrionales | Desulfovibrionaceae | *Desulfovibrio* | 30569 |
| OTU316 | Bacteroidetes | Bacteroidia | Bacteroidales | Bacteroidaceae | *Bacteroides uniformis* | 1733364 |
| OTU317 | Firmicutes | Erysipelotrichi | Erysipelotrichales | Erysipelotrichaceae |  | 814989 |
| OTU318 | Firmicutes | Clostridia | Clostridiales | Eubacteriaceae | *Pseudoramibacter-Eubacterium* | 109633 |
| OTU319 | Bacteroidetes | Bacteroidia | Bacteroidales | Bacteroidaceae | *Bacteroides uniformis* | 4376964 |
| OTU320 | Firmicutes | Clostridia | Clostridiales | Ruminococcaceae |  | 509101 |
| OTU321 | Firmicutes | Clostridia | Clostridiales | Ruminococcaceae |  | New.ReferenceOTU7 |
| OTU322 | Firmicutes | Clostridia | Clostridiales | Lachnospiraceae |  | New.ReferenceOTU23 |
| OTU323 | Bacteroidetes | Bacteroidia | Bacteroidales | [Odoribacteraceae] | *Butyricimonas* | 988375 |
| OTU324 | Firmicutes | Clostridia | Clostridiales | Lachnospiraceae |  | New.ReferenceOTU225 |
| OTU325 | Firmicutes | Clostridia | Clostridiales | Lachnospiraceae | *[Ruminococcus]* | 182245 |
| OTU326 | Bacteroidetes | Bacteroidia | Bacteroidales | Bacteroidaceae | *Bacteroides* | 4406925 |
| OTU327 | Tenericutes | Mollicutes | RF39 |  |  | New.ReferenceOTU260 |
| OTU328 | Firmicutes | Clostridia | Clostridiales | Lachnospiraceae |  | New.ReferenceOTU281 |
| OTU329 | Bacteroidetes | Bacteroidia | Bacteroidales | Bacteroidaceae | *Bacteroides* | New.ReferenceOTU158 |
| OTU330 | Firmicutes | Clostridia | Clostridiales | Ruminococcaceae |  | 581079 |
| OTU331 | Bacteroidetes | Bacteroidia | Bacteroidales | Bacteroidaceae | *Bacteroides* | 583117 |
| OTU332 | Firmicutes | Clostridia | Clostridiales | Lachnospiraceae |  | New.ReferenceOTU318 |
| OTU333 | Bacteroidetes | Bacteroidia | Bacteroidales | Porphyromonadaceae | *Parabacteroides distasonis* | 4442459 |
| OTU334 | Firmicutes | Clostridia | Clostridiales | Lachnospiraceae | *Dorea* | 4178726 |
| OTU335 | Firmicutes | Clostridia | Clostridiales | Ruminococcaceae |  | New.ReferenceOTU235 |
| OTU336 | Firmicutes | Erysipelotrichi | Erysipelotrichales | Erysipelotrichaceae | *[Eubacterium] dolichum* | 584949 |
| OTU337 | Firmicutes | Clostridia | Clostridiales | Ruminococcaceae |  | New.ReferenceOTU263 |
| OTU338 | Bacteroidetes | Bacteroidia | Bacteroidales | Bacteroidaceae | *Bacteroides* | 180141 |
| OTU339 | Proteobacteria | Deltaproteobacteria | Desulfovibrionales | Desulfovibrionaceae |  | 113417 |
| OTU340 | Firmicutes | Clostridia | Clostridiales | Lachnospiraceae |  | New.ReferenceOTU191 |
| OTU341 | Proteobacteria | Betaproteobacteria | Burkholderiales | Oxalobacteraceae | *Oxalobacter formigenes* | 360508 |
| OTU342 | Firmicutes | Erysipelotrichi | Erysipelotrichales | Erysipelotrichaceae | *[Eubacterium] dolichum* | New.ReferenceOTU275 |
| OTU343 | Bacteroidetes | Bacteroidia | Bacteroidales | Bacteroidaceae | *Bacteroides uniformis* | 584541 |
| OTU344 | Firmicutes | Clostridia | Clostridiales | Ruminococcaceae | *Oscillospira* | 266841 |
| OTU345 | Firmicutes | Erysipelotrichi | Erysipelotrichales | Erysipelotrichaceae |  | 216989 |
| OTU346 | Firmicutes | Clostridia | Clostridiales | Ruminococcaceae | *Oscillospira* | 548692 |
| OTU347 | Firmicutes | Clostridia | Clostridiales |  |  | 158576 |
| OTU348 | Bacteroidetes | Bacteroidia | Bacteroidales | Porphyromonadaceae | *Parabacteroides* | New.ReferenceOTU91 |
| OTU349 | Firmicutes | Erysipelotrichi | Erysipelotrichales | Erysipelotrichaceae | *Coprobacillus* | 233953 |
| OTU350 | Firmicutes | Clostridia | Clostridiales | Ruminococcaceae | *Oscillospira* | 198753 |
| OTU351 | Bacteroidetes | Bacteroidia | Bacteroidales | Rikenellaceae |  | New.ReferenceOTU29 |
| OTU352 | Firmicutes | Clostridia | Clostridiales | Ruminococcaceae | *Oscillospira* | 548032 |
| OTU353 | Bacteroidetes | Bacteroidia | Bacteroidales | Rikenellaceae |  | 556472 |
| OTU354 | Firmicutes | Clostridia | Clostridiales | Lachnospiraceae | *Blautia producta* | New.ReferenceOTU74 |
| OTU355 | Firmicutes | Clostridia | Clostridiales | Ruminococcaceae |  | New.ReferenceOTU142 |
| OTU356 | Bacteroidetes | Bacteroidia | Bacteroidales | [Odoribacteraceae] | *Butyricimonas* | 525344 |
| OTU357 | Firmicutes | Erysipelotrichi | Erysipelotrichales | Erysipelotrichaceae |  | 849723 |
| OTU358 | Firmicutes | Clostridia | Clostridiales | Lachnospiraceae |  | 2148365 |
| OTU359 | Firmicutes | Clostridia | Clostridiales | Lachnospiraceae |  | New.ReferenceOTU159 |
| OTU360 | Firmicutes | Clostridia | Clostridiales |  |  | 358944 |
| OTU361 | Firmicutes | Clostridia | Clostridiales | Ruminococcaceae | *Ruminococcus* | 175619 |
| OTU362 | Firmicutes | Clostridia | Clostridiales | Lachnospiraceae |  | 574122 |
| OTU363 | Firmicutes | Clostridia | Clostridiales | Lachnospiraceae |  | 157516 |
| OTU364 | Bacteroidetes | Bacteroidia | Bacteroidales | Bacteroidaceae | *Bacteroides* | New.ReferenceOTU66 |
| OTU365 | Firmicutes | Clostridia | Clostridiales | Lachnospiraceae | *[Ruminococcus]* | 592901 |
| OTU366 | Firmicutes | Clostridia | Clostridiales |  |  | 4133460 |
| OTU367 | Firmicutes | Clostridia | Clostridiales | Ruminococcaceae | *Oscillospira* | 204093 |
| OTU368 | Firmicutes | Clostridia | Clostridiales | Lachnospiraceae | *Dorea* | New.ReferenceOTU110 |
| OTU369 | Firmicutes | Clostridia | Clostridiales | Lachnospiraceae | *[Ruminococcus]* | New.ReferenceOTU77 |
| OTU370 | Firmicutes | Clostridia | Clostridiales | Lachnospiraceae | *[Ruminococcus]* | 129692 |
| OTU371 | Firmicutes | Clostridia | Clostridiales |  |  | New.ReferenceOTU109 |
| OTU372 | Firmicutes | Clostridia | Clostridiales | Ruminococcaceae | *Ruminococcus* | 716984 |
| OTU373 | Bacteroidetes | Bacteroidia | Bacteroidales | Porphyromonadaceae | *Parabacteroides* | 4308591 |
| OTU374 | Firmicutes | Clostridia | Clostridiales | Ruminococcaceae | *Oscillospira* | 564334 |
| OTU375 | Bacteroidetes | Bacteroidia | Bacteroidales | Rikenellaceae |  | 574644 |
| OTU376 | Firmicutes | Clostridia | Clostridiales | Lachnospiraceae |  | New.ReferenceOTU56 |
| OTU377 | Firmicutes | Clostridia | Clostridiales | Ruminococcaceae |  | New.ReferenceOTU227 |
| OTU378 | Firmicutes | Clostridia | Clostridiales |  |  | 360890 |
| OTU379 | Bacteroidetes | Bacteroidia | Bacteroidales | Porphyromonadaceae | *Parabacteroides* | New.ReferenceOTU156 |
| OTU380 | Proteobacteria | Deltaproteobacteria | Desulfovibrionales | Desulfovibrionaceae |  | 263518 |
| OTU381 | Bacteroidetes | Bacteroidia | Bacteroidales | Bacteroidaceae | *Bacteroides* | 198449 |
| OTU382 | Fusobacteria | Fusobacteriia | Fusobacteriales | Fusobacteriaceae | *Fusobacterium* | 3461438 |
| OTU383 | Firmicutes | Clostridia | Clostridiales |  |  | 192385 |
| OTU384 | Bacteroidetes | Bacteroidia | Bacteroidales | Bacteroidaceae | *Bacteroides* | 351292 |
| OTU385 | Firmicutes | Clostridia | Clostridiales | Ruminococcaceae | *Ruminococcus* | 1654480 |
| OTU386 | Bacteroidetes | Bacteroidia | Bacteroidales | Bacteroidaceae | *Bacteroides* | 1698008 |
| OTU387 | Proteobacteria | Deltaproteobacteria | Desulfovibrionales | Desulfovibrionaceae | *Bilophila* | 841907 |
| OTU388 | Firmicutes | Clostridia | Clostridiales | Ruminococcaceae |  | 307680 |
| OTU389 | Verrucomicrobia | Verrucomicrobiae | Verrucomicrobiales | Verrucomicrobiaceae | *Akkermansia muciniphila* | 899714 |
| OTU390 | Firmicutes | Clostridia | Clostridiales | Lachnospiraceae |  | New.ReferenceOTU24 |
| OTU391 | Bacteroidetes | Bacteroidia | Bacteroidales | Bacteroidaceae | *Bacteroides ovatus* | 187443 |
| OTU392 | Firmicutes | Clostridia | Clostridiales | Ruminococcaceae | *Oscillospira* | 335550 |
| OTU393 | Firmicutes | Clostridia | Clostridiales |  |  | 197427 |
| OTU394 | Firmicutes | Clostridia | Clostridiales |  |  | 561607 |
| OTU395 | Proteobacteria | Betaproteobacteria | Burkholderiales | Alcaligenaceae | *Sutterella* | 4366834 |
| OTU396 | Firmicutes | Clostridia | Clostridiales |  |  | New.ReferenceOTU60 |
| OTU397 | Bacteroidetes | Bacteroidia | Bacteroidales | [Odoribacteraceae] | *Odoribacter* | New.ReferenceOTU311 |
| OTU398 | Firmicutes | Clostridia | Clostridiales | Ruminococcaceae |  | 271290 |
| OTU399 | Firmicutes | Clostridia | Clostridiales | Ruminococcaceae |  | 397444 |
| OTU400 | Actinobacteria | Coriobacteriia | Coriobacteriales | Coriobacteriaceae | *Slackia* | 560981 |
| OTU401 | Firmicutes | Clostridia | Clostridiales | Ruminococcaceae |  | 309433 |
| OTU402 | Firmicutes | Clostridia | Clostridiales | Veillonellaceae | *Phascolarctobacterium* | 916143 |
| OTU403 | Firmicutes | Clostridia | Clostridiales | Lachnospiraceae |  | 189299 |
| OTU404 | Bacteroidetes | Bacteroidia | Bacteroidales | [Paraprevotellaceae] | *Paraprevotella* | 1106254 |
| OTU405 | Firmicutes | Clostridia | Clostridiales | Eubacteriaceae | *Pseudoramibacter-Eubacterium* | New.ReferenceOTU307 |
| OTU406 | Firmicutes | Clostridia | Clostridiales |  |  | 157693 |
| OTU407 | Bacteroidetes | Bacteroidia | Bacteroidales | Bacteroidaceae | *Bacteroides* | 270094 |
| OTU408 | Bacteroidetes | Bacteroidia | Bacteroidales | [Barnesiellaceae] |  | 850905 |
| OTU409 | Actinobacteria | Coriobacteriia | Coriobacteriales | Coriobacteriaceae |  | 295768 |
| OTU410 | Firmicutes | Clostridia | Clostridiales | Ruminococcaceae |  | 586525 |
| OTU411 | Bacteroidetes | Bacteroidia | Bacteroidales | Rikenellaceae |  | 171772 |
| OTU412 | Firmicutes | Clostridia | Clostridiales |  |  | 1105486 |
| OTU413 | Cyanobacteria | 4C0d-2 | YS2 |  |  | 320915 |
| OTU414 | Bacteroidetes | Bacteroidia | Bacteroidales | Porphyromonadaceae | *Parabacteroides* | New.ReferenceOTU258 |
| OTU415 | Cyanobacteria | 4C0d-2 | YS2 |  |  | New.ReferenceOTU85 |
| OTU416 | Firmicutes | Clostridia | Clostridiales | Lachnospiraceae |  | New.ReferenceOTU64 |
| OTU417 | Bacteroidetes | Bacteroidia | Bacteroidales | Bacteroidaceae | *Bacteroides* | 194670 |
| OTU418 | Firmicutes | Clostridia | Clostridiales |  |  | New.ReferenceOTU21 |
| OTU419 | Bacteroidetes | Bacteroidia | Bacteroidales | Bacteroidaceae | *Bacteroides ovatus* | 4428185 |
| OTU420 | Firmicutes | Clostridia | Clostridiales | Lachnospiraceae |  | New.ReferenceOTU233 |
| OTU421 | Bacteroidetes | Bacteroidia | Bacteroidales | Rikenellaceae |  | New.ReferenceOTU224 |
| OTU422 | Firmicutes | Clostridia | Clostridiales | Lachnospiraceae |  | New.ReferenceOTU255 |
| OTU423 | Firmicutes | Clostridia | Clostridiales |  |  | 187350 |
| OTU424 | Actinobacteria | Coriobacteriia | Coriobacteriales | Coriobacteriaceae | *Eggerthella lenta* | 849361 |
| OTU425 | Firmicutes | Clostridia | Clostridiales | Ruminococcaceae |  | New.ReferenceOTU220 |
| OTU426 | Firmicutes | Clostridia | Clostridiales | Ruminococcaceae |  | New.ReferenceOTU86 |
| OTU427 | Firmicutes | Clostridia | Clostridiales | Lachnospiraceae |  | 187812 |
| OTU428 | Firmicutes | Clostridia | Clostridiales | Ruminococcaceae | *Anaerotruncus* | 311961 |
| OTU429 | Firmicutes | Clostridia | Clostridiales | Ruminococcaceae | *Oscillospira* | 585227 |
| OTU430 | Tenericutes | Mollicutes | RF39 |  |  | New.ReferenceOTU49 |
| OTU431 | Actinobacteria | Coriobacteriia | Coriobacteriales | Coriobacteriaceae |  | 742358 |
| OTU432 | Firmicutes | Clostridia | Clostridiales | Lachnospiraceae |  | New.ReferenceOTU301 |
| OTU433 | Bacteroidetes | Bacteroidia | Bacteroidales | Bacteroidaceae | *Bacteroides* | 184174 |
| OTU434 | Cyanobacteria | 4C0d-2 | YS2 |  |  | New.ReferenceOTU99 |
| OTU435 | Firmicutes | Clostridia | Clostridiales | Lachnospiraceae | *Blautia* | New.ReferenceOTU154 |
| OTU436 | Firmicutes | Clostridia | Clostridiales | Ruminococcaceae |  | 208927 |
| OTU437 | Firmicutes | Clostridia | Clostridiales | Ruminococcaceae |  | New.ReferenceOTU73 |
| OTU438 | Firmicutes | Clostridia | Clostridiales | Lachnospiraceae |  | 3175741 |
| OTU439 | Firmicutes | Clostridia | Clostridiales | Christensenellaceae |  | 363519 |
| OTU440 | Firmicutes | Clostridia | Clostridiales | Lachnospiraceae |  | New.ReferenceOTU32 |
| OTU441 | Bacteroidetes | Bacteroidia | Bacteroidales | Porphyromonadaceae | *Parabacteroides* | 522582 |
| OTU442 | Firmicutes | Clostridia | Clostridiales | Lachnospiraceae |  | New.ReferenceOTU30 |
| OTU443 | Firmicutes | Clostridia | Clostridiales | Ruminococcaceae | *Ruminococcus* | 199543 |
| OTU444 | Cyanobacteria | 4C0d-2 | YS2 |  |  | New.ReferenceOTU200 |
| OTU445 | Proteobacteria | Alphaproteobacteria | RF32 |  |  | 4299126 |
| OTU446 | Bacteroidetes | Bacteroidia | Bacteroidales | Bacteroidaceae | *Bacteroides fragilis* | 2921213 |
| OTU447 | Firmicutes | Clostridia | Clostridiales | Ruminococcaceae | *Ruminococcus* | 4456702 |
| OTU448 | Firmicutes | Clostridia | Clostridiales | Lachnospiraceae | *Coprococcus* | New.ReferenceOTU199 |
| OTU449 | Firmicutes | Clostridia | Clostridiales | Ruminococcaceae |  | New.ReferenceOTU111 |
| OTU450 | Bacteroidetes | Bacteroidia | Bacteroidales | Bacteroidaceae | *Bacteroides* | 175844 |
| OTU451 | Proteobacteria | Gammaproteobacteria | Enterobacteriales | Enterobacteriaceae | *Proteus* | 470879 |
| OTU452 | Firmicutes | Clostridia | Clostridiales | Ruminococcaceae |  | 414089 |
| OTU453 | Firmicutes | Clostridia | Clostridiales | Ruminococcaceae |  | 205241 |
| OTU454 | Bacteroidetes | Bacteroidia | Bacteroidales | Rikenellaceae |  | 323400 |
| OTU455 | Firmicutes | Clostridia | Clostridiales | Eubacteriaceae | *Pseudoramibacter-Eubacterium* | 2576209 |
| OTU456 | Firmicutes | Clostridia | Clostridiales | Christensenellaceae |  | 4410097 |
| OTU457 | Firmicutes | Clostridia | Clostridiales | Lachnospiraceae |  | New.ReferenceOTU219 |
| OTU458 | Bacteroidetes | Bacteroidia | Bacteroidales | Bacteroidaceae | *Bacteroides* | New.ReferenceOTU51 |
| OTU459 | Tenericutes | Mollicutes | RF39 |  |  | New.ReferenceOTU100 |
| OTU460 | Proteobacteria | Alphaproteobacteria | RF32 |  |  | New.ReferenceOTU168 |
| OTU461 | Firmicutes | Clostridia | Clostridiales | Lachnospiraceae | *Blautia producta* | New.ReferenceOTU226 |
| OTU462 | Firmicutes | Clostridia | Clostridiales |  |  | 111282 |
| OTU463 | Firmicutes | Clostridia | Clostridiales |  |  | 194417 |
| OTU464 | Firmicutes | Clostridia | Clostridiales | Ruminococcaceae |  | 393504 |
| OTU465 | Firmicutes | Clostridia | Clostridiales | Ruminococcaceae | *Ruminococcus* | 361702 |
| OTU466 | Firmicutes | Clostridia | Clostridiales | Christensenellaceae | *Christensenella* | 217109 |
| OTU467 | Firmicutes | Clostridia | Clostridiales | Lachnospiraceae |  | 1848553 |
| OTU468 | Bacteroidetes | Bacteroidia | Bacteroidales | Rikenellaceae |  | 234443 |
| OTU469 | Bacteroidetes | Bacteroidia | Bacteroidales | Bacteroidaceae | *Bacteroides* | New.ReferenceOTU94 |
| OTU470 | Firmicutes | Clostridia | Clostridiales | Ruminococcaceae |  | 315223 |
| OTU471 | Firmicutes | Erysipelotrichi | Erysipelotrichales | Erysipelotrichaceae | *Coprobacillus* | 4396248 |
| OTU472 | Firmicutes | Clostridia | Clostridiales | Lachnospiraceae | *[Ruminococcus]* | New.ReferenceOTU70 |
| OTU473 | Bacteroidetes | Bacteroidia | Bacteroidales | Rikenellaceae |  | 4331760 |
| OTU474 | Firmicutes | Clostridia | Clostridiales | Ruminococcaceae | *Ruminococcus* | 298535 |
| OTU475 | Firmicutes | Clostridia | Clostridiales | Ruminococcaceae |  | 381219 |
| OTU476 | Firmicutes | Clostridia | Clostridiales | Lachnospiraceae | *Dorea* | 180227 |
| OTU477 | Firmicutes | Clostridia | Clostridiales | Lachnospiraceae | *Blautia* | 189998 |
| OTU478 | Firmicutes | Clostridia | Clostridiales | Ruminococcaceae | *Oscillospira* | 369389 |
| OTU479 | Firmicutes | Clostridia | Clostridiales | Veillonellaceae |  | 166334 |
| OTU480 | Proteobacteria | Alphaproteobacteria | Rhizobiales | Brucellaceae | *Ochrobactrum* | 345813 |
| OTU481 | Firmicutes | Clostridia | Clostridiales | Lachnospiraceae |  | 583089 |
| OTU482 | Actinobacteria | Coriobacteriia | Coriobacteriales | Coriobacteriaceae |  | New.ReferenceOTU262 |
| OTU483 | Firmicutes | Clostridia | Clostridiales |  |  | 849147 |
| OTU484 | Firmicutes | Clostridia | Clostridiales | Lachnospiraceae | *Dorea* | 174471 |
| OTU485 | Firmicutes | Clostridia | Clostridiales | Ruminococcaceae |  | 359120 |
| OTU486 | Firmicutes | Clostridia | Clostridiales | Ruminococcaceae |  | 311732 |
| OTU487 | Firmicutes | Erysipelotrichi | Erysipelotrichales | Erysipelotrichaceae | *[Eubacterium] dolichum* | 587530 |
| OTU488 | Firmicutes | Clostridia | Clostridiales | Ruminococcaceae |  | New.ReferenceOTU246 |
| OTU489 | Firmicutes | Clostridia | Clostridiales | Ruminococcaceae |  | 197442 |
| OTU490 | Tenericutes | Mollicutes | RF39 |  |  | 517044 |
| OTU491 | Bacteroidetes | Bacteroidia | Bacteroidales | Porphyromonadaceae | *Parabacteroides* | 4441084 |
| OTU492 | Firmicutes | Clostridia | Clostridiales | Ruminococcaceae | *Oscillospira* | 264932 |
| OTU493 | Actinobacteria | Coriobacteriia | Coriobacteriales | Coriobacteriaceae |  | New.ReferenceOTU65 |
| OTU494 | Firmicutes | Clostridia | Clostridiales | Ruminococcaceae |  | 198618 |
| OTU495 | Firmicutes | Clostridia | Clostridiales |  |  | 343238 |
| OTU496 | Firmicutes | Clostridia | Clostridiales | Lachnospiraceae | *[Ruminococcus]* | 185972 |
| OTU497 | Firmicutes | Clostridia | Clostridiales | Ruminococcaceae |  | 191754 |
| OTU498 | Firmicutes | Clostridia | Clostridiales | Ruminococcaceae | *Ruminococcus* | New.ReferenceOTU176 |
| OTU499 | Firmicutes | Clostridia | Clostridiales | Ruminococcaceae |  | 147100 |
| OTU500 | Firmicutes | Clostridia | Clostridiales | Ruminococcaceae | *Ruminococcus* | 187386 |
| OTU501 | Firmicutes | Clostridia | Clostridiales | Lachnospiraceae | *[Ruminococcus]* | 241224 |
| OTU502 | Firmicutes | Clostridia | Clostridiales | Ruminococcaceae |  | 352243 |
| OTU503 | Firmicutes | Clostridia | Clostridiales | Ruminococcaceae | *Ruminococcus* | 213870 |
| OTU504 | Firmicutes | Clostridia | Clostridiales | Christensenellaceae | *Christensenella* | 177179 |
| OTU505 | Bacteroidetes | Bacteroidia | Bacteroidales | Bacteroidaceae | *Bacteroides* | New.ReferenceOTU229 |
| OTU506 | Proteobacteria | Betaproteobacteria | Burkholderiales | Alcaligenaceae | *Sutterella* | 2470914 |
| OTU507 | Firmicutes | Clostridia | Clostridiales |  |  | New.ReferenceOTU147 |
| OTU508 | Bacteroidetes | Bacteroidia | Bacteroidales | Porphyromonadaceae | *Parabacteroides* | New.ReferenceOTU101 |
| OTU509 | Firmicutes | Clostridia | Clostridiales | Ruminococcaceae |  | 712388 |
| OTU510 | Firmicutes | Clostridia | Clostridiales | Lachnospiraceae |  | 512682 |
| OTU511 | Firmicutes | Clostridia | Clostridiales | Eubacteriaceae | *Pseudoramibacter_Eubacterium* | 4464292 |
| OTU512 | Proteobacteria | Epsilonproteobacteria | Campylobacterales | Helicobacteraceae | *Flexispira* | 311173 |
| OTU513 | Actinobacteria | Coriobacteriia | Coriobacteriales | Coriobacteriaceae |  | 175508 |
| OTU514 | Firmicutes | Clostridia | Clostridiales | Lachnospiraceae | *[Ruminococcus]* | 199555 |
| OTU515 | Firmicutes | Clostridia | Clostridiales | Ruminococcaceae | *Oscillospira* | 247557 |
| OTU516 | Firmicutes | Clostridia | Clostridiales | Lachnospiraceae |  | New.ReferenceOTU264 |
| OTU517 | Firmicutes | Clostridia | Clostridiales | Lachnospiraceae | *[Ruminococcus]* | New.ReferenceOTU47 |
| OTU518 | Firmicutes | Clostridia | Clostridiales |  |  | 2657412 |
| OTU519 | Firmicutes | Clostridia | Clostridiales | Lachnospiraceae | *[Ruminococcus] gnavus* | New.ReferenceOTU283 |
| OTU520 | Firmicutes | Bacilli | Lactobacillales | Streptococcaceae | *Lactococcus* | 303204 |
| OTU521 | Bacteroidetes | Bacteroidia | Bacteroidales | Bacteroidaceae | *Bacteroides fragilis* | 2380160 |
| OTU522 | Bacteroidetes | Bacteroidia | Bacteroidales | [Barnesiellaceae] |  | New.ReferenceOTU285 |
| OTU523 | Firmicutes | Clostridia | Clostridiales | Lachnospiraceae |  | 188145 |
| OTU524 | Firmicutes | Clostridia | Clostridiales | Ruminococcaceae |  | 4456590 |
| OTU525 | Cyanobacteria | 4C0d-2 | YS2 |  |  | 4405128 |
| OTU526 | Firmicutes | Clostridia | Clostridiales | Ruminococcaceae | *Oscillospira* | 4356307 |
| OTU527 | Firmicutes | Clostridia | Clostridiales | Ruminococcaceae |  | 177697 |
| OTU528 | Bacteroidetes | Bacteroidia | Bacteroidales | Rikenellaceae |  | 216599 |
| OTU529 | Firmicutes | Clostridia | Clostridiales | Ruminococcaceae | *Oscillospira* | 260322 |
| OTU530 | Proteobacteria | Betaproteobacteria | Burkholderiales | Alcaligenaceae | *Sutterella* | 179261 |
| OTU531 | Firmicutes | Clostridia | Clostridiales | Ruminococcaceae |  | New.ReferenceOTU82 |
| OTU532 | Spirochaetes | [Brachyspirae] | [Brachyspirales] | Brachyspiraceae | *Brachyspira* | 83206 |
| OTU533 | Bacteroidetes | Bacteroidia | Bacteroidales | Rikenellaceae | *Rikenella* | 569030 |
| OTU534 | Firmicutes | Erysipelotrichi | Erysipelotrichales | Erysipelotrichaceae | *Coprobacillus* | 369763 |
| OTU535 | Firmicutes | Clostridia | Clostridiales |  |  | 581658 |
| OTU536 | Firmicutes | Clostridia | Clostridiales | Ruminococcaceae |  | 232828 |
| OTU537 | Bacteroidetes | Bacteroidia | Bacteroidales | Rikenellaceae |  | 199190 |
| OTU538 | Firmicutes | Clostridia | Clostridiales | Ruminococcaceae | *Oscillospira* | 422283 |
| OTU539 | Proteobacteria | Alphaproteobacteria | RF32 |  |  | 211720 |
| OTU540 | Firmicutes | Clostridia | Clostridiales |  |  | New.ReferenceOTU177 |
| OTU541 | Bacteroidetes | Bacteroidia | Bacteroidales | Rikenellaceae | *Rikenella* | New.ReferenceOTU306 |
| OTU542 | Firmicutes | Clostridia | Clostridiales | Lachnospiraceae |  | 189331 |
| OTU543 | Bacteroidetes | Bacteroidia | Bacteroidales | Porphyromonadaceae | *Parabacteroides* | 846127 |
| OTU544 | Firmicutes | Clostridia | Clostridiales | Clostridiaceae |  | New.ReferenceOTU310 |
| OTU545 | Firmicutes | Clostridia | Clostridiales | Lachnospiraceae | *[Ruminococcus] gnavus* | New.ReferenceOTU309 |
| OTU546 | Actinobacteria | Coriobacteriia | Coriobacteriales | Coriobacteriaceae | *Eggerthella lenta* | New.ReferenceOTU316 |
| OTU547 | Firmicutes | Clostridia | Clostridiales | Ruminococcaceae | *Ruminococcus* | 362765 |
| OTU548 | Firmicutes | Clostridia | Clostridiales | Lachnospiraceae | *[Ruminococcus]* | New.ReferenceOTU261 |
| OTU549 | Cyanobacteria | 4C0d-2 | YS2 |  |  | New.ReferenceOTU181 |
| OTU550 | Firmicutes | Clostridia | Clostridiales | Ruminococcaceae | *Oscillospira* | 364341 |
| OTU551 | Bacteroidetes | Bacteroidia | Bacteroidales | Porphyromonadaceae | *Parabacteroides* | New.ReferenceOTU272 |
| OTU552 | Verrucomicrobia | Verrucomicrobiae | Verrucomicrobiales | Verrucomicrobiaceae | *Akkermansia muciniphila* | 359376 |
| OTU553 | Bacteroidetes | Bacteroidia | Bacteroidales | Bacteroidaceae | *Bacteroides* | New.ReferenceOTU197 |
| OTU554 | Lentisphaerae | [Lentisphaeria] | Victivallales | Victivallaceae |  | 2136916 |
| OTU555 | Tenericutes | Mollicutes | RF39 |  |  | New.ReferenceOTU116 |
| OTU556 | Firmicutes | Clostridia | Clostridiales | Lachnospiraceae | *[Ruminococcus] gnavus* | 330564 |
| OTU557 | Firmicutes | Clostridia | Clostridiales |  |  | New.ReferenceOTU203 |
| OTU558 | Firmicutes | Clostridia | Clostridiales | Ruminococcaceae |  | 196249 |
| OTU559 | Firmicutes | Clostridia | Clostridiales | Lachnospiraceae |  | 183867 |
| OTU560 | Bacteroidetes | Bacteroidia | Bacteroidales | Bacteroidaceae | *Bacteroides* | 2487129 |
| OTU561 | Firmicutes | Clostridia | Clostridiales | Ruminococcaceae |  | 594206 |
| OTU562 | Firmicutes | Clostridia | Clostridiales | Ruminococcaceae |  | 759816 |
| OTU563 | Firmicutes | Erysipelotrichi | Erysipelotrichales | Erysipelotrichaceae | *[Eubacterium] dolichum* | New.ReferenceOTU34 |
| OTU564 | Bacteroidetes | Bacteroidia | Bacteroidales | Rikenellaceae |  | 213671 |
| OTU565 | Firmicutes | Clostridia | Clostridiales | Lachnospiraceae |  | 4447176 |
| OTU566 | Tenericutes | Mollicutes | RF39 |  |  | 1100992 |
| OTU567 | Bacteroidetes | Bacteroidia | Bacteroidales | Bacteroidaceae | *Bacteroides fragilis* | 3506423 |
| OTU568 | Firmicutes | Clostridia | Clostridiales | Ruminococcaceae |  | 198031 |
| OTU569 | Actinobacteria | Actinobacteria | Bifidobacteriales | Bifidobacteriaceae | *Bifidobacterium* | New.ReferenceOTU245 |
| OTU570 | Bacteroidetes | Bacteroidia | Bacteroidales | Bacteroidaceae | *Bacteroides* | 4301298 |
| OTU571 | Firmicutes | Clostridia | Clostridiales | Clostridiaceae |  | 330714 |
| OTU572 | Firmicutes | Clostridia | Clostridiales | Eubacteriaceae | *Anaerofustis* | 527988 |
| OTU573 | Bacteroidetes | Bacteroidia | Bacteroidales | Bacteroidaceae | *Bacteroides* | 589277 |
| OTU574 | Firmicutes | Clostridia | Clostridiales | Ruminococcaceae |  | 1094991 |
| OTU575 | Proteobacteria | Epsilonproteobacteria | Campylobacterales | Helicobacteraceae | *Helicobacter* | 1135946 |
| OTU576 | Bacteroidetes | Bacteroidia | Bacteroidales | Bacteroidaceae | *Bacteroides* | 363625 |
| OTU577 | Firmicutes | Clostridia | Clostridiales | Ruminococcaceae |  | 566155 |
| OTU578 | Bacteroidetes | Bacteroidia | Bacteroidales | [Barnesiellaceae] |  | 199354 |
| OTU579 | Firmicutes | Clostridia | Clostridiales | Lachnospiraceae | *Coprococcus* | New.ReferenceOTU319 |
| OTU580 | Firmicutes | Clostridia | Clostridiales |  |  | 368025 |
| OTU581 | Firmicutes | Clostridia | Clostridiales | Ruminococcaceae |  | 544859 |
| OTU582 | Firmicutes | Clostridia | Clostridiales | Ruminococcaceae | *Faecalibacterium prausnitzii* | 201658 |
| OTU583 | Firmicutes | Clostridia | Clostridiales | Ruminococcaceae |  | 221429 |
| OTU584 | Fusobacteria | Fusobacteriia | Fusobacteriales | Fusobacteriaceae | *Fusobacterium* | 572889 |
| OTU585 | Firmicutes | Clostridia | Clostridiales | Ruminococcaceae | *Oscillospira* | 186460 |
| OTU586 | Actinobacteria | Coriobacteriia | Coriobacteriales | Coriobacteriaceae | *Adlercreutzia* | 631764 |
| OTU587 | Cyanobacteria | 4C0d-2 | YS2 |  |  | New.ReferenceOTU216 |
| OTU588 | Firmicutes | Bacilli |  |  |  | 99508 |
| OTU589 | Firmicutes | Clostridia | Clostridiales | Lachnospiraceae |  | New.ReferenceOTU221 |
| OTU590 | Bacteroidetes | Bacteroidia | Bacteroidales | Bacteroidaceae | *Bacteroides uniformis* | 589071 |
| OTU591 | Proteobacteria | Gammaproteobacteria | Aeromonadales | Succinivibrionaceae |  | 851937 |
| OTU592 | Firmicutes | Clostridia | Clostridiales | Ruminococcaceae |  | 349802 |
| OTU593 | Firmicutes | Clostridia | Clostridiales | Ruminococcaceae | *Faecalibacterium prausnitzii* | 158553 |
| OTU594 | Actinobacteria | Coriobacteriia | Coriobacteriales | Coriobacteriaceae |  | 2423305 |
| OTU595 | Firmicutes | Clostridia | Clostridiales | Ruminococcaceae |  | New.ReferenceOTU172 |
| OTU596 | Firmicutes | Clostridia | Clostridiales | Ruminococcaceae | *Oscillospira* | 184084 |
| OTU597 | Firmicutes | Clostridia | Clostridiales | Ruminococcaceae |  | New.ReferenceOTU228 |
| OTU598 | Bacteroidetes | Bacteroidia | Bacteroidales | Bacteroidaceae | *Bacteroides* | New.ReferenceOTU217 |
| OTU599 | Actinobacteria | Actinobacteria | Bifidobacteriales | Bifidobacteriaceae | *Bifidobacterium* | New.ReferenceOTU105 |
| OTU600 | Firmicutes | Clostridia | Clostridiales | Lachnospiraceae | *[Ruminococcus]* | New.ReferenceOTU244 |
| OTU601 | Firmicutes | Clostridia | Clostridiales | Veillonellaceae | *Veillonella* | New.ReferenceOTU107 |
| OTU602 | Firmicutes | Clostridia | Clostridiales | Lachnospiraceae | *Blautia* | New.ReferenceOTU71 |


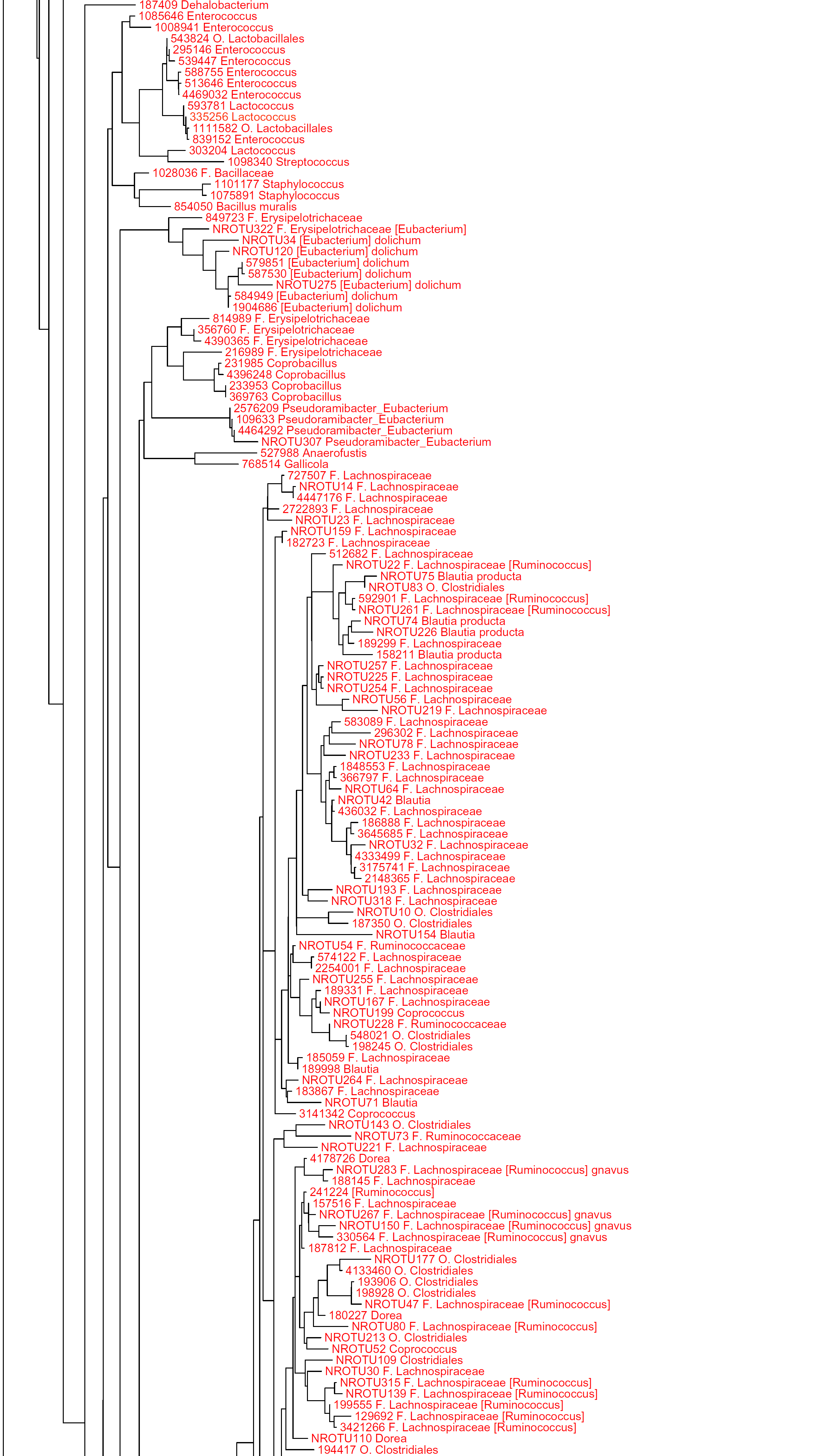


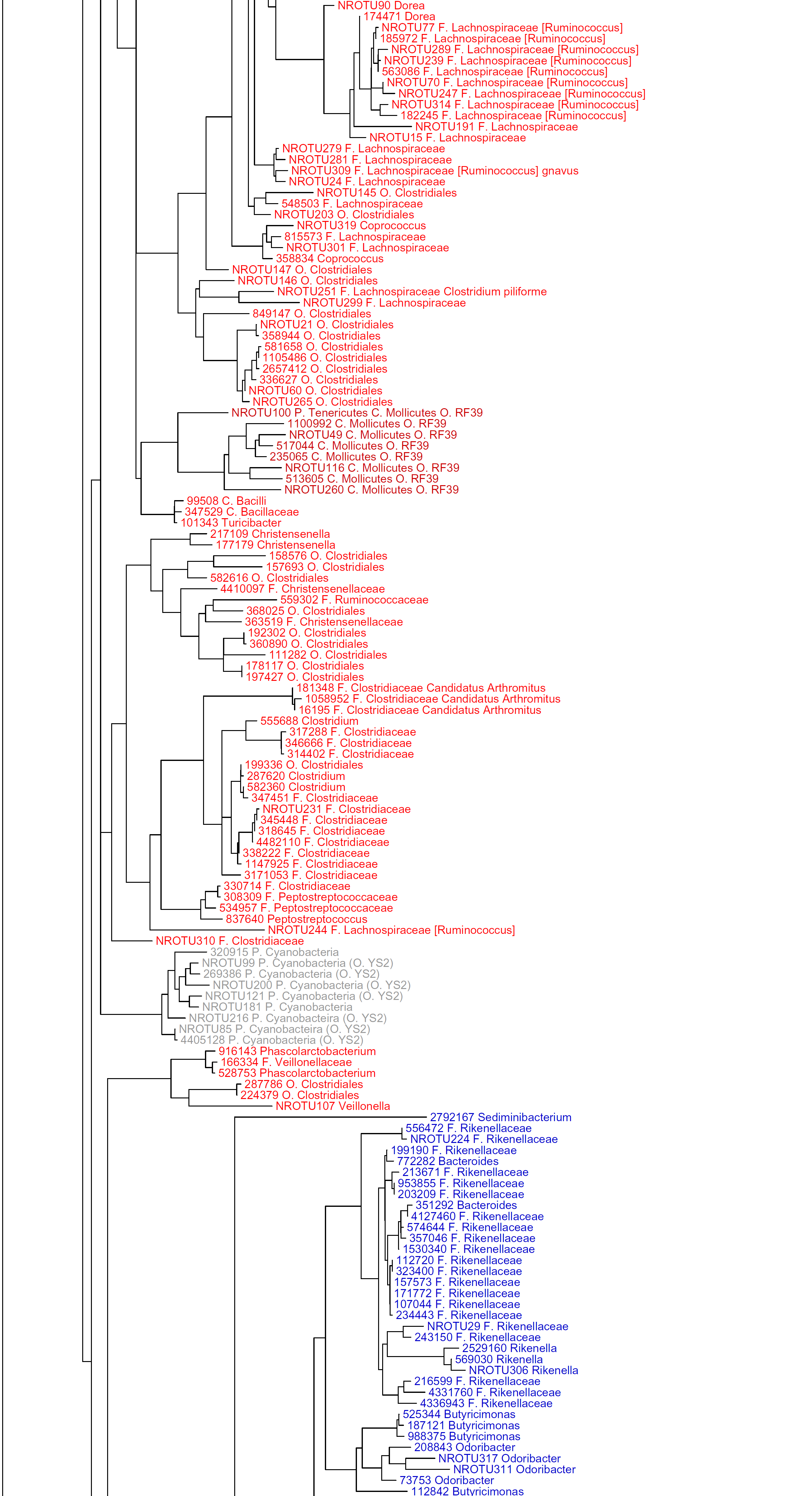


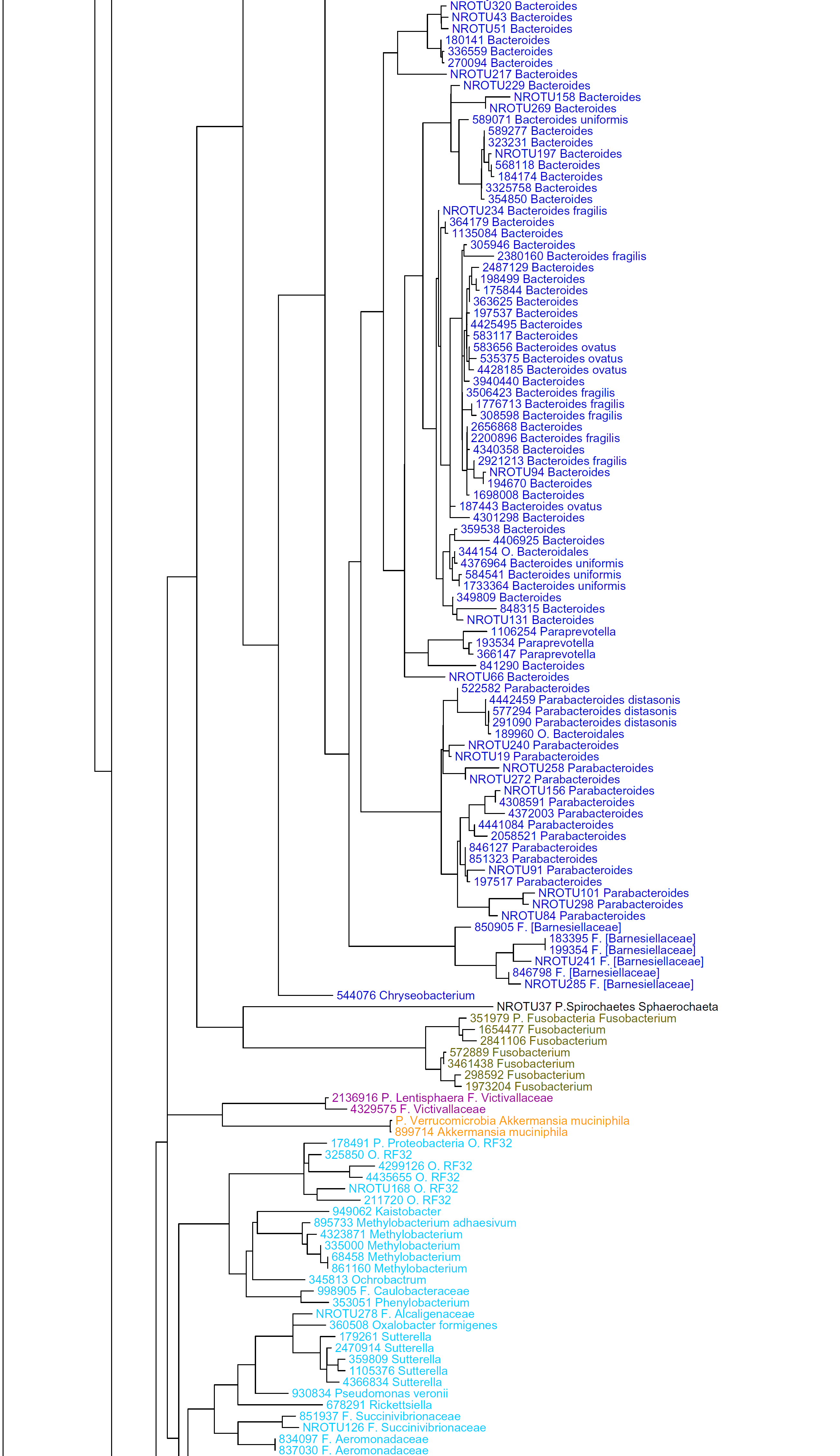


**Figure S1.** Tree reflecting the among-OTUs phylogenetic relationships for OTUs found in cloacae of great-spotted cuckoos and magpies. Tip labels include the Illumina label and the identification of the OTU at the deepest level with available information.
